# *fbl*-Typing of *Staphylococcus lugdunensis*: A Frontline Tool for Epidemiological Studies, but Not Predictive of Fibrinogen Binding Ability

**DOI:** 10.3389/fmicb.2019.01109

**Published:** 2019-05-17

**Authors:** Sandrine Dahyot, Jérémie Lebeurre, Floriane Laumay, Xavier Argemi, Charline Dubos, Ludovic Lemée, Gilles Prévost, Patrice François, Martine Pestel-Caron

**Affiliations:** ^1^UNIROUEN, GRAM EA2656, Rouen University Hospital, Normandie Université, Rouen, France; ^2^UNIROUEN, GRAM EA2656, Normandie Université, Rouen, France; ^3^Genomic Research Laboratory, Service of Infectious Diseases, University of Geneva Hospitals, Geneva, Switzerland; ^4^VBP EA7290, Fédération de Médecine Translationnelle de Strasbourg, Institut de Bactériologie, Centre Hospitalier Régional Universitaire, Université de Strasbourg, Strasbourg, France; ^5^Maladies Infectieuses et Tropicales, Hôpitaux Universitaires de Strasbourg, Strasbourg, France

**Keywords:** *Staphylococcus lugdunensis*, *fbl*-typing, epidemiology, phylogeny, fibrinogen adhesion

## Abstract

*Staphylococcus lugdunensis* is increasingly recognized as a potent pathogen, responsible for severe infections with an outcome resembling that of *Staphylococcus aureus*. Here, we developed and evaluated a tool for *S. lugdunensis* typing, using DNA sequence analysis of the repeat-encoding region (R-domain) in the gene encoding the fibrinogen (Fg)-binding protein Fbl (*fbl*-typing). We typed 240 *S. lugdunensis* isolates from various clinical and geographical origins. The length of the R-domain ranged from 9 to 52 repeats. *fbl*-typing identified 54 unique 18-bp repeat sequences and 92 distinct *fbl*-types. The discriminatory power of *fbl*-typing was higher than that of multilocus sequence typing (MLST) and equivalent to that of tandem repeat sequence typing. *fbl*-types could assign isolates to MLST clonal complexes with excellent predictive power. The ability to promote adherence to immobilized human Fg was evaluated for 55 isolates chosen to reflect the genetic diversity of the *fbl* gene. We observed no direct correlation between Fg binding ability and *fbl*-types. However, the lowest percentage of Fg binding was observed for isolates carrying a 5′-end frameshift mutation of the *fbl* gene and for those harboring fewer than 43 repeats in the R-domain. qRT-PCR assays for some isolates revealed no correlation between *fbl* gene expression and Fg binding capacity. In conclusion, this study shows that *fbl*-typing is a useful tool in *S. lugdunensis* epidemiology, especially because it is an easy, cost-effective, rapid and portable method (http://fbl-typing.univ-rouen.fr/). The impact of *fbl* polymorphism on the structure of the protein, its expression on the cell surface and in virulence remains to be determined.

## Introduction

*Staphylococcus lugdunensis* is a member of the coagulase-negative staphylococci (CoNS). It belongs to the normal human skin flora, colonizing several distinct niches such as perineal and inguinal areas (Bieber and Kahlmeter, 2010) but is increasingly recognized as a potent human pathogen (Frank et al., 2008). The behavior of *S. lugdunensis* is similar in many ways to that of *Staphylococcus aureus*, exhibiting higher virulence than other CoNS (Frank et al., 2008). *S. lugdunensis* can cause various types of infections, ranging from localized to systemic diseases (Zinkernagel et al., 2008; Heldt Manica and Cohen, 2017). It has mainly been reported in skin and soft tissue infections (Papapetropoulos et al., 2013), but it is also responsible for catheter-related bloodstream infections, bone and joint infections (Argemi et al., 2017) and severe infective endocarditis (Non and Santos, 2017). Unlike *S. aureus*, very few pathogenicity factors have been characterized (Heilbronner et al., 2011). Some *in vitro* studies have suggested the existence of several virulence factors, including hemolysins, adhesion proteins [Fg-binding protein Fbl (Mitchell et al., 2004) and von Willebrand factor-binding protein vWbl (Nilsson et al., 2004b)] and iron-regulated surface determinant proteins (Heilbronner et al., 2016).

A variety of molecular typing methods have been developed for *S. lugdunensis* characterization, including pulsed-field gel electrophoresis (Yeh et al., 2015), MLST (Chassain et al., 2012), and MVLST (Didi et al., 2014). Phylogenetic analyses by MLST and MVLST have shown the clonal population structure, the mutational evolution of this pathogen, and the absence of hypervirulent lineages. MLST has become the method of choice for unambiguous clonal definition (Aanensen and Spratt, 2005), however, there is a need for a portable tool with greater discriminatory power than MLST for micro-evolution based epidemiology.

Another method involves the typing of multiple, rapidly evolving *loci* containing repeated sequences, known as VNTRs. These markers can be used to build allelic profiles in MLVA systems, in order to discriminate between clonal bacterial populations (Van Belkum et al., 2007). Recently, we developed the first two VNTR-based schemes for *S. lugdunensis* typing: a classic length-based MLVA method and a sequence-based MLVA method known as the TRST method (Dahyot et al., 2018). These typing methods were more discriminating than MLST and MVLST, and represent promising tools for molecular epidemiological studies of *S. lugdunensis*.

A more practical alternative to the multilocus approach is the sequence-based typing of one or two highly polymorphic *loci*. Indeed, when analyzing clonal populations, phylogenetic inferences are rarely confounded *via* homologous genetic recombination (Zaiß et al., 2009). This approach was previously developed for a number of pathogens, including *S. aureus* (*spa*) (Shopsin et al., 1999), *Streptococcus pyogenes* (*emm*) (Beall et al., 1996), and *Clostridium difficile* (TR6, TR10) (Zaiß et al., 2009). Currently, *S. aureus* protein A (*spa*) gene is one of the most widely used single genetic markers for *S. aureus* typing (Asadollahi et al., 2018). *spa*-typing is an established method based on the evaluation of number and sequence variation in mainly 24-bp repeats at the X-region of *spa* (Frenay et al., 1996). *spa*-typing was shown to be highly concordant with other typing methods such as MLST or pulsed-field gel electrophoresis (Shopsin et al., 1999; Harmsen et al., 2003; Golding et al., 2008). Due to the clonal population structure of *S. aureus, spa*-typing is considered as a highly discriminating method for outbreak investigation and for the assignment of strains to phylogenetic lineages in population studies (Koreen et al., 2004; Strommenger et al., 2008). Like *spa*-typing, a method using the *clf*A R-domain (18-pb repeats) of clumping factor ClfA was shown to be useful for *S. aureus* typing and grouping host-specific lineages (Said et al., 2009, 2010). This repeat region acts as a flexible stalk to extend the Fg binding domain from the cell surface (Hartford et al., 1997). The *clfA* R-domain varies in size among different *S. aureus* strains (McDevitt and Foster, 1995; Said et al., 2009). Moreover, repeat copy number has been shown to affect adherence and clumping titers of *S. aureus* strains (Hartford et al., 1997; Risley et al., 2007).

**FIGURE 1:**
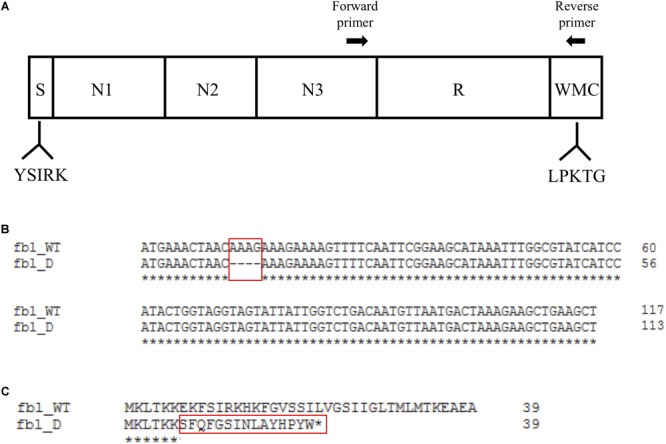
Schematic representation of the Fbl protein and alignments of the 5′-end region. **(A)** The different regions indicated are S (signal peptide), N1-, N2-, and N3-domains, R (repeat domain) and WMC (W, wall spanning; M, membrane spanning; C, cytoplasmic positively charged tail). Conserved motifs are indicated below. The locations of the forward and reverse primers used to amplify and sequence the R-domain are shown at the top. **(B)** Nucleotide sequence alignment of the first 117 bp of *fbl* of isolates exhibiting the wild type sequence (fbl_WT) and isolates with a 4 bp deletion (fbl_D). Numbering begins at the start codon. Identical sequences are denoted by asterisks. The position of the 4 bp deletion is shown with a red box. Alignment was obtained using the Clustal Omega tool https://www.ebi.ac.uk/Tools/msa/clustalo/. **(C)** Wild type (fbl_WT) and truncated Fbl (fbl_D) translation products of the signal peptide region of Fbl (amino acids 1–39). Beneath the alignment, preserved amino acid are indicated with asterisks. The deletion causing the frameshift mutation (E7fs) is indicated with a red box. The asterisk denotes the stop codon (L22STOP).

Interestingly, the Fbl protein coded by the *fbl* gene of *S. lugdunensis* is closely related to the ClfA of *S. aureus*, showing 62% amino acid identity in the Fg-binding region (Mitchell et al., 2004; Nilsson et al., 2004a). However, the R-domain of Fbl (SDSDSA hexapeptide motif) is slightly different to that of ClfA (SD repeats only). It is encoded by a variable number of 18-bp repeats located immediately upstream of the region coding the C-terminal cell wall attachment sequence (Figure 1A). The size of the R-domain of *fbl* varies according to the strains (Mitchell et al., 2004). In this context, the analysis of the *fbl* R-domain could be a potential useful marker for *S. lugdunensis* typing.

Therefore, the objectives of this study were (i) to develop and evaluate the use of an *fbl* R-domain repeat-based system to type diverse *S. lugdunensis* isolates, (ii) to compare its typing performance with MLST and TRST, and (iii) to determine the potential impact of *fbl* polymorphism on the ability of isolates to bind to Fg *in vitro*.

## Materials and Methods

### Bacterial Isolates

A total of 240 *S. lugdunensis* human isolates recovered from 230 patients were used in this study (Supplementary Table S1). Among them, ten pairs of isolates were collected from ten patients at time intervals ranging from 0 to 17 days. A first panel, the “TRST panel,” included 128 *S. lugdunensis* isolates previously characterized by MLST and TRST and collected from five regions of France and from Sweden (Dahyot et al., 2018). A further set of 106 clinical isolates recovered from 101 patients was collected at Rouen University Hospital in France from January 2016 to December 2016. Lastly, six clinical isolates whose complete genome sequence was available in our laboratory were included in this study. Overall, the 240 isolates were recovered from skin and soft tissue infections (*n* = 50), bone and joint infections (*n* = 41), deep infections (*n* = 22), bacteremia (*n* = 12), endocarditis (*n* = 9), medical device infections (*n* = 8), other infections (*n* = 12), carriage (*n* = 79), and from undocumented origin (*n* = 7). All 240 isolates were identified by matrix-assisted laser desorption/ionization time-of-flight mass.

### DNA Extraction and PCR Amplification

Isolates were grown overnight at 37°C on tryptic soy agar with 5% horse blood agar plate. DNA was extracted using the InstaGene Matrix kit (Bio-Rad, Marnes-la-Coquette, France) according to the manufacturer’s recommendations. PCRs were performed on a Veriti Thermal Cycler (Applied Biosystems, Foster City, CA, United States) in a final volume of 25 μl containing 12.5 μl GoTaq G2 Green Master Mix (Promega, Charbonnières-Les-Bains, France), 0.50 μM of each primer and 5 μl of DNA.

### PCR Amplification and DNA Sequencing of the *fbl* Gene

The *fbl* gene of the 15 genomes of *S. lugdunensis* available in the GenBank database and of six complete genomes sequenced by our laboratory was aligned using clustal multiple sequence alignment^1^. Primers fbl_R_F and fbl_R_R were designed for the *fbl*-typing assay development to flank the *fbl* R-domain (Table 1 and Figure 1A). Primers fbl_N_F and fbl_N_R were designed in the 5′ part of the gene to amplify and sequence a 264 bp fragment corresponding to the region upstream the *fbl* gene and its first 125 bp (Table 1). All primers were designed using OLIGO Primer Design software (Molecular Biology Insights, United States). Sequences were BLAST checked for homologous regions. The PCR conditions were as follows: denaturation at 95°C for 5 min, followed by 35 cycles of amplification including denaturation at 95°C for 30 s, annealing at 55°C for 30 s, and extension at 72°C for 30 s. A final extension step at 72°C for 5 min was performed. All PCR products were separated on 1% agarose gel by electrophoresis, purified, and sequenced in both forward and reverse directions by Eurofins GATC Biotech SARL (Konstanz, Germany).

**Table 1:** Primers used in this study.

	Name of
Target	primer	Primer sequence (5′–3′)	References
*fbl* R-domain amplification and sequencing by conventional PCR	fbl_R_F fbl_R_R	TGGCATGGGATAATGAAGTAGCCACTCCCGTATAGTAACT	This study
*fbl* Nter-domain amplification and sequencing by conventional PCR	fbl_N_F fbl_N_R	TTGCCTGTATAATGCTATCTTTTTGACAAATTACGCTCCx	This study
*fbl* gene expression by qRT-PCR	fbl_qRT_F fbl_qRT_R	AATAGCGAGGCACAAGCAATAGCAGGTGATACATCGGTGG	This study
16S rRNA gene expression by qRT-PCR	Q3 Q4	GAGGAAGGIGIGGAIGACGTAGICCCGIGAACGTATTCAC	Tseng et al., 2003

### Identification of *fbl*-Types and Cluster Analysis

The number and the sequence of *fbl* repeats were determined with BioNumerics software (Version 7.6, Applied Maths, Sint-Martens-Latem, Belgium) using the “polymorphic VNTR typing” plugin. To check that the entire repeat array had been sequenced, the flanking sequences were spotted but were not included as typing information. Each unique 18-bp repeat sequence was identified by a numeric code, known as the “repeat code.” Each unique combination of repeat sequences defined an “*fbl*-type,” with a number followed by a lowercase letter, where the number corresponds to the number of repeats and the letter to the combination of the different repeat sequences, e.g., fbl42a. Hence, each *fbl*-type denotes a collection of specific repeat units arranged in a precise pattern.

Cluster analysis of *fbl* sequences was performed using the “polymorphic VNTR typing” plugin of BioNumerics software. With this plugin, sequences are compared and aligned using an algorithm based on the DSI (duplications, substitutions, and indels) model for pairwise alignment of repeats (Benson, 1997). A minimum spanning tree was generated from the similarity matrix with the root node assigned to the ST with the greatest number of related types. Default software parameters were used for analysis with a bin distance of 1%, i.e., two entries having a similarity of 99–100% will have a distance of 0 and two entries that have a similarity of 98–99% will have a distance of 1. For cluster analysis, only *fbl*-types separated by a minimum spanning tree distance of ≤2 (i.e., if they were ≥97% similar) were considered closely related and assigned to the same cluster.

### Data Analysis

The discriminatory ability and the congruence of the results obtained using the different typing methods were evaluated. Simpson’s diversity index (DI) (Hunter and Gaston, 1988) and AWs (Severiano et al., 2011) [plus CIs as described by Grundmann et al. (2001)] were calculated using the online tool at http://www.comparingpartitions.info/?link=Tool. If the CI of any two methods overlaps, they may have similar discriminatory powers at a 95% confidence level. The ratio of non-synonymous to synonymous substitutions (*d_N_*/*d_S_*) was calculated with MEGA version 7 software using the Maximum Likelihood analysis of natural selection codon-by-codon. A ratio of >1 indicates positive selection, a ratio of 1 indicates no selection pressure and a ratio of <1 indicates purifying selection.

### Test of *fbl* Stability

To determine the stability of the *fbl* R-domain, two isolates were subcultured on tryptic soy agar for 30 consecutive days by streaking a single colony from each isolate on agar plates. The original culture and subcultures 10, 20, 30 were used for DNA extraction and the total DNA was subjected to the *fbl* assay.

### Construction and Validation of an *fbl*-Typing Server

An *fbl* allele database was created to store all identified *fbl*-types in FASTA format. Perfect identity hits report the corresponding *fbl*-type whereas imperfect hits report an “unknown” *fbl*-type, and the user is encouraged to contact the curator to update the database with this new variant. The Web tool has been made publicly available and is hosted by Rouen Normandy University^2^. Individual FASTA assemblies of the paired Sanger sequences of 92 isolates identified with BioNumerics software were analyzed with this newly developed Web tool, and output results were compared.

### Rapid Slide Latex Agglutination Tests

A rapid latex agglutination test was performed on all isolates using the commercial *S. aureus* agglutination kit Pastorex^TM^ Staph Plus (Bio-Rad, Marnes-la-Coquette, France) according to the manufacturer’s instructions.

### Adherence of Bacterial Cells to Immobilized Fibrinogen

Bacterial adhesion to solid phase-adsorbed human Fg was assessed in a 96-well plate format. Flat-bottom microtiter plates were coated for 1 h at 37°C with 50 μl per well of 10 μg/ml human Fg or PBS. The plates were washed three times with PBS and incubated at 37°C for 1 h after addition of 250 μl of 2% human serum albumin. The plates were washed three times with PBS prior to the inoculation of bacterial suspension prepared as follows. Bacterial isolates were inoculated in Mueller Hinton broth and incubated overnight at 37°C under constant agitation. A volume of 100 μl of suspension was inoculated in fresh broth (10 ml) and incubated at 37°C for 3 h. Bacteria were washed twice with PBS and adjusted to an OD_600 nm_ = 0.9. Fifty microliters of adjusted bacterial suspension were added per well and after 1 h 30 min of incubation at 37°C, the wells were rinsed three times with PBS. Adherent bacteria were fixed for 30 min at 60°C, stained with 95 μl of 0.5% crystal violet in 0.5% ethanol for 15 min and air-dried after washings. Crystal violet staining was solubilized into 100 μl of dimethyl sulfoxide and the absorbance measured at OD_595 nm_ using a microplate reader. Adherence of isolates was evaluated in at least five wells, and the experiment was performed at least twice. *S. aureus* DU5925-mutant of the main Fg-binding adhesin was used as a negative control (non-adherent); *S. aureus* 8325-4 and 8325-4 pCF4 were used as positive controls in all experiments (adherent and hyper-adherent, respectively). Adherence of isolates was expressed relative to that of 8325-4 (100%). All results were expressed as mean percentages ± standard error of mean. After suppression of significant outliers (*P* < 0.05, online tool^3^), adherence of isolates was compared to that of DU5925 using Dunnett’s multiple comparisons test, and the global difference was analyzed using ordinary one-way ANOVA (GraphPad Prism statistical analysis). When the percentage of binding to solid-phase Fg was significantly different to that of the negative control strain it was categorized as “+++” and when it was lower it was interpreted as “-”. Strains that did not belong to the two previous categories were considered as “+”. The impact of *fbl* genetic diversity (presence of the 5′-end frameshift mutation or number of repeats in the R-domain) on adherence was evaluated using a non-parametric analysis of variance test (Kruskal–Wallis) on R software (version 3.5.1). A *P*-value of <0.05 was regarded as significant.

### Total RNA Extraction

Cells were grown in trypticase soy broth at 37°C in a shaking incubator set at 150 rpm to mid-exponential phase (OD_600 nm_ = 1), and 1 ml was centrifuged (5 min at 8,000 × *g*, 4°C). Pellets were resuspended in 200 μl TE Buffer (10 mM Tris–HCl pH 8 and 1 mM EDTA) containing 2.1 mg lysozyme (Sigma-Aldrich, St. Louis, MO, United States) and 10 μg lysostaphin (Sigma-Aldrich, St. Louis, MO, United States). Samples were vortexed for 30 s, incubated at 37°C for 10 min, and lysed using the RA1 lysis buffer provided in the NucleoSpin^®^ RNA kit (Macherey Nagel, Hoerdt, France) supplemented with β-mercaptoethanol (Sigma-Aldrich, St. Louis, MO, United States). Next, a bead-beating procedure alternating 1 min cycles of beating with incubation on ice was performed. RNA was isolated using the NucleoSpin^®^ RNA kit according to the manufacturer’s instructions. The amount of RNA yielded was assessed with a NanoDrop spectrophotometer (Thermo Fisher Scientific, Montigny-le-Bretonneux, France). RNA samples were then treated by TURBO DNA-*free*^TM^ kit (Ambion, Austin, TX, United States) according to the manufacturer’s instructions.

### Quantitative Real-Time PCR

The cDNA synthesis was performed using the Omniscript^®^ RT kit (Qiagen, Hilden, Germany) according to the manufacturer’s instructions. A total of 2 μg of RNA was reverse transcribed in a 20 μL volume reaction using random primers (Promega, Madison, WI, United States) and RNAse inhibitor (40 U/μl) (Invitrogen, Carlsbad, CA, United States). The quantitative RT-PCR (qRT-PCR) primers listed in Table 1 were used in 20 μl PCR mixtures which included SYBR^®^ Green PCR Master Mix (Bio-Rad, Marnes la Coquette, France) and 1 μl of diluted cDNA template (1:10 for *fbl* and 1:100 for 16S rRNA). A CFX96 real-time PCR detection system (Bio-Rad, Marnes la Coquette, France) was used for qRT-PCR with the following PCR amplification program: initial denaturation step at 95°C for 10 min, followed by 40 cycles of amplification including denaturation at 95°C for 30 s, annealing at 55°C for 30 s, and extension at 72°C for 30 s. Melting curve analysis was used to identify specific products increasing in 0.5°C increment every 5 s from 55 to 95°C. Relative *fbl* expression levels for each isolate were quantified using the gene expression analysis module of CFX Manager^TM^ software (2^-Δ^*^C^*^T^ method), with 16S rRNA gene as reference to normalize the results. All *S. lugdunensis* isolates were assayed as triplicates in each experimental run, and three independent biological assays were performed. The statistical significance of *fbl* gene expression differences between the groups of Fg-binding-negative (-) and Fg-binding-positive (+++) *S. lugdunensis* isolates was determined based on Mann–Whitney–Wilcoxon measurements. A *P*-value of < 0.05 was regarded as significant.

## Results

### *fbl* R-Domain Polymorphism

Two hundred and forty *S. lugdunensis* isolates from various clinical and geographical backgrounds were characterized by *fbl*-typing. The sequencing and assembling of all repeats was successful, irrespective of length. The number of repeats varied between 9 and 52, as identified by sequencing PCR products ranging from 586 to 1360 bp in length. The mean number of repeats in R-domain was 42 per isolate. Sequence analysis identified 54 unique 18-bp repeat sequences, each given a numeric code (Supplementary Table S2). The deduced amino acid sequences of the repeats allowed identification of 13 types of 6-amino-acid units (Supplementary Table S2). The most frequent pattern was DSDSDA. Polymorphism resulted in synonymous substitutions for most repeats, with a ratio of non-synonymous to synonymous substitutions of 0.045, suggesting a potential role of environmental selective pressure in their evolution.

### *fbl*-Typing Results

The organization of the repeats of the *fbl* R-domain (composition, number and order of repeats) from each of the isolates was represented as an *fbl*-type repeat code. Ninety-two distinct *fbl*-types were defined for the 240 isolates, designated fbl9a to fbl52a. Supplementary Table S3 provides a complete overview of these profiles. The most common *fbl*-types were fbl47b (*n* = 43), fbl45f (*n* = 24) and fbl41a (*n* = 13). There were 68 unique *fbl*-types (represented by only one patient). *fbl*-type was not accurately predicted by the length of the variable region, as the number of repeats was the same for many of the types (for example, 15 unique *fbl*-types had 45 repeat elements). To assess the discriminatory power of *fbl*-typing, we included only one isolate per patient as all the isolate pairs had the same *fbl*-type. Simpson’s DI calculated from the 230 remaining unrelated isolates was 0.946 (Table 2).

**Table 2:** Discriminatory power of the three typing methods for unrelated isolates.

	All isolates (*n* = 230)			TRST panel (*n* = 123)		
Typing	No. of			No. of
method	genotypes	DI^a^	CI^b^ 95%	genotypes	DI^a^	CI^b^ 95%
*fbl*-typing	92	0.946	0.929–0.964	60	0.964	0.949–0.979
MLST	*ND*^c^	*ND*^c^	*ND*^c^	25	0.899	0.872–0.926
TRST	*ND*^c^	*ND*^c^	*ND*^c^	69	0.943	0.915–0.971

*^*a*^DI, Simpson’s diversity index. ^*b*^CI, confidence interval. ^*c*^*ND*, not determined.*

### *fbl* Clustering

**FIGURE 2:**
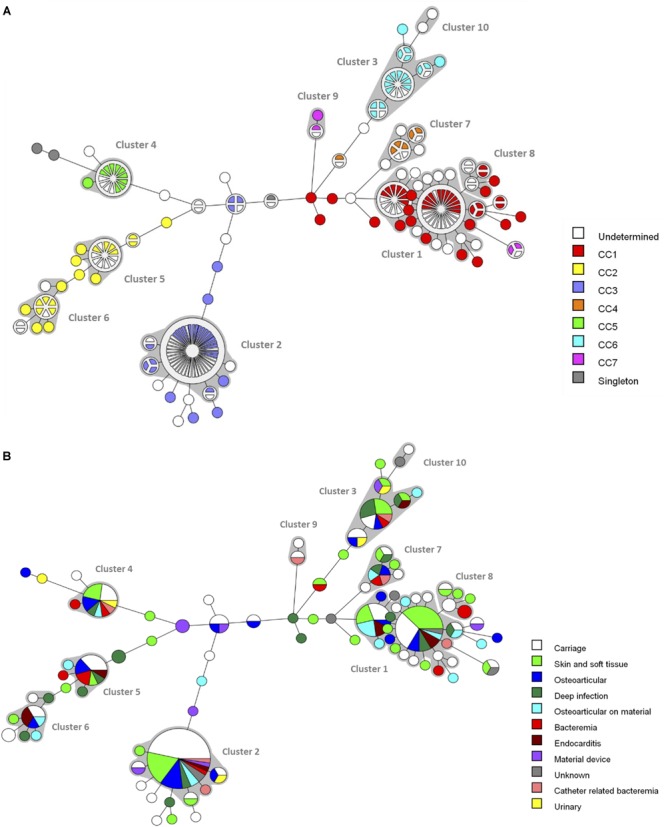
Minimum spanning tree analysis of the 240 *S. lugdunensis* isolates based on *fbl*-types. Cluster analysis was performed using the polymorphic VNTR typing plugin of BioNumerics. *fbl*-types separated by a minimum spanning tree distance of ≤2 (i.e., if they were ≥97% similar) were considered closely related and assigned to the same cluster. Each circle represents an *fbl*-type and its size is proportional to the number of isolates. The length of the branches expressed the minimum spanning tree distance between two *fbl*-types. Gray zones around circles delineate *fbl* clusters. The colors used are based **(A)** on clonal complexes (CCs) defined by MLST, undetermined, unknown CC (isolates non-characterized by MLST) and **(B)** on clinical contexts.

A minimum spanning tree was constructed for the 240 isolates in order to visualize the relationships among the *fbl*-types (Figure 2A). The 92 unique *fbl*-types were distributed over 10 clusters (cluster 1–10), each containing more than 2 isolates, and 46 singletons. *fbl* clustering allowed the grouping of isolates that had similar repeat organizations. The main cluster was cluster 1 (57 isolates) consisting of 20 *fbl*-types, then cluster 2 (52 isolates) with 6 *fbl*-types. These two largest *fbl* clusters comprised 45% of all the isolates. Eighty-one % of the 240 isolates analyzed in the study were part of a cluster.

The distribution of the isolates failed to identify any correlation between *fbl*-types and clinical settings (Figure 2B).

### Discriminatory Power and Concordance Between Typing Methods

*fbl*-typing results were compared with those of MLST and TRST for 128 isolates representative of the genetic diversity of *S. lugdunensis* (Dahyot et al., 2018). Twenty-five STs belonging to 7 CCs, 69 TRTs, and 60 *fbl*-types were identified for these isolates (Table 2). Simpson’s DI for 123 of these 128 isolates (only one genotype was included per patient to avoid any bias) was calculated at 0.964 for *fbl*-typing, 0.899 for MLST and 0.943 for TRST (Table 2). Thus, MLST showed the lowest discriminatory power, whereas *fbl*-typing and TRST showed comparable high discriminatory power. The five pairs of epidemiologically related isolates considered as clonal by MLST and TRST belonged to the same *fbl*-types. This supports the *in vivo* stability of the *fbl* R-domain, and attests the epidemiological concordance of the method.

The clustering by both *fbl*-typing and MLST was similar, as shown by the minimum spanning tree analysis (Figure 2A). For example, isolates of *fbl* clusters 1 and 2 belonged to CC1 and CC3, respectively. One of the most noticeable findings was that each *fbl*-type was associated with only a single CC. Thus, *fbl*-typing provided CC assessment for all the isolates (Supplementary Table S3). This was confirmed by the calculation of the AW of *fbl*-typing (Table 3). If any two isolates shared the same *fbl*-type, they had a 100% probability of belonging to the same CC. Supplementary Table S3 is provided as a preliminary guideline for correspondence between *fbl*-types and MLST CCs.

**Table 3:** Adjusted Wallace coefficients and 95% confidence intervals for the 123 unrelated isolates typed by multilocus sequence typing (MLST), tandem repeat sequence typing (TRST), and *fbl*-typing.

	CC (MLST)	ST (MLST)	TRST type	*fbl*-type
CC		0.477	0.257	0.158
(MLST)		(0.398–0.555)	(0.176–0.339)	(0.089–0.226)
ST	1.000		0.487	0.225
(MLST)	(1.000–1.000)		(0.371–0.602)	(0.126–0.324)
TRST type	1.000	0.901		0.215
	(1.000–1.000)	(0.828–0.974)		(0.076–0.353)
*fbl*-type	1.000	0.680	0.351	
	(1.000–1.000)	(0.521–0.840)	(0.202–0.500)	

*CC, clonal complex; ST, sequence type.*

However, *fbl*-typing was less able to predict ST (AW = 0.680) (Table 3), as an *fbl*-type could correspond to several STs. Conversely, some STs could contain up to 11 different *fbl*-types, like ST2 and ST3. On the other hand, the low AW coefficients between *fbl*-typing and TRST showed that *fbl*-types was able to predict TRTs with a 35% probability (Table 3).

Therefore, *fbl*-typing further discriminated new genotypes in the MLST CCs on multiple occasions (for six of the seven CCs analyzed) (Table 4). For instance, 12 *fbl*-types were identified for the CC2 isolates whereas only five STs and five TRTs were described for these isolates by MLST and TRST, respectively. Isolates of CC1 encompassed 20 unique *fbl*-types while MLST only distinguished 5 STs whereas TRST allowed the identification of 28 TRTs.

**Table 4:** Ability of the three typing methods to discriminate the 123 unrelated isolates within major *S. lugdunensis* clonal complexes (CCs) defined by multilocus sequence typing (MLST).

CC	MLST		TRST		*fbl*-typing	
(No. of	No. of		No. of		No. of
isolates)	STs	DI^a^	TRTs	DI^a^	*fbl*-types	DI^a^
1 (37)	5	0.752	28	0.962	20	0.896
2 (18)	5	0.405	5	0.405	12	0.922
3 (30)	3	0.131	6	0.310	11	0.710
4 (6)	3	0.600	6	1	3	0.733
5 (10)	3	0.511	8	0.956	2	0.200
6 (15)	2	0.133	10	0.914	6	0.810
7 (4)	2	0.667	3	0.833	3	0.833
Singleton (3)	2	0.667	3	1	3	1
Total	25	0.899	69	0.943	60	0.964

*ST, sequence type; TRT, tandem repeat sequence typing type. ^*a*^DI, Simpson’s diversity index.*

### Stability of *fbl* R-Domain

The stability of the *fbl* R-domain was examined, showing that, for each isolate, *fbl*-types were identical between the original isolates and their 10th, 20th, and 30th subcultures, indicating that the R-domain of *fbl* was stable.

### Construction and Validation of an “*fbl*-Typing Server”

An *fbl*-typing server was constructed to perform *fbl*-typing using sequencing data saved in a FASTA format. It is a free access Web-based service hosted by Rouen Normandy University (see text footnote 2). The server contains data on the 92 *fbl*-types available at the time of the study. The *fbl*-typing Web tool was evaluated and identified *fbl*-types correctly with a 100% identity match.

### *fbl* 5′-End Polymorphism

We examined the complete sequence variation of the *fbl* gene in published *S. lugdunensis* genomes, and discovered a 4 bp deletion occurring at position 12 from the start codon for two isolates (Figure 1B). *In silico* analysis suggested that this deletion caused a frameshift mutation leading to a premature translational stop (L22STOP) (Figure 1C). One hundred and forty-six isolates were screened for this deletion by Sanger sequencing. The deletion was found in 27 isolates, belonging to different *fbl*-types (Supplementary Table S1). Twenty-six of the 27 isolates belonged to CC1, with a distribution that varied according to the ST. All isolates characterized as ST12 (*n* = 8) and ST15 (*n* = 6) had this deletion, whereas isolates belonging to ST1 (*n* = 12) and ST7 (*n* = 1) did not. Within ST6 (CC1), 11 of the 15 isolates (73%) had the deletion. The only isolate that had the deletion and was not from CC1 belonged to ST3 (CC3).

### Fibrinogen Binding

The 240 isolates were first tested with rapid latex agglutination test which yielded positive results for 77 isolates (32%) (Table 5). Then, the ability to bind to immobilized human Fg was tested for 55 isolates with different *fbl* gene characteristics in terms of *fbl*-types and presence or absence of the 5′-end deletion. The level of bacterial binding to immobilized Fg varied considerably between the clinical isolates tested (0 to 133% of the positive control strain) (Supplementary Figure S1). Six of the 55 isolates significantly bound to solid-phase Fg (+++) and ten adhered poorly (+) (Table 5). Thirty-nine isolates did not bind to solid-phase Fg (-).

**Table 5:** Bacterial adherence to immobilized fibrinogen.

Clinical		Premature stop		Binding to solid-	Agglutination	
isolate	*fbl*-type	codon in *fbl* gene	MLST CC	phase fibrinogen	test	Clinical source	City
SL_T29	43g	–	1	+++^∗∗∗∗^	Positive	Material device infection	Rouen
SL_R30	45l	–	*ND*	+++^∗∗^	Positive	SSTI	Rouen
SL_V03	47b	–	3	+++^∗∗^	Positive	SSTI	Strasbourg
SL_V74	45b	–	2	+++^∗^	Positive	Bacteremia	Strasbourg
SL_T117	45f	–	1	+++^∗^	Positive	Carriage	Kronoberg
SL_V51	45h	–	1	+++^∗^	Positive	BJI	Strasbourg
SL_T118	40b	–	6	+	Negative	Carriage	Kronoberg
SL_T122	43g	–	1	+	Positive	Carriage	Tours
SL_V06	45c	–	2	+	Negative	Deep infection	Strasbourg
SL_R34	45f	–	*ND*	+	Positive	SSTI	Rouen
SL_V05	45g	–	1	+	Positive	Deep infection	Strasbourg
SL_V35	45i	–	1	+	Positive	Catheter related bacteremia	Strasbourg
SL_R44	47b	–	*ND*	+	Negative	Carriage	Rouen
SL_V34	52a	–	4	+	Positive	SSTI	Strasbourg
SL_V07	21a	L22STOP	1	+	Negative	Deep infection	Strasbourg
SL_V24	44a	L22STOP	1	+	Negative	BJI on material	Strasbourg
SL_C47	13a	–	3	–	Negative	Carriage	Strasbourg
SL_R59	13a	–	*ND*	–	Negative	BJI	Rouen
SL_R87	18a	–	*ND*	–	Negative	Carriage	Rouen
SL_V50	24a	–	3	–	Negative	BJI on material	Strasbourg
SL_R74	37a	–	*ND*	–	Negative	Carriage	Rouen
SL_DSM	41b	–	6	–	Positive	SSTI	Unknown
SL_T55	41b	–	6	–	Negative	SSTI	Nantes
SL_V70	41c	–	7	–	Negative	SSTI	Strasbourg
SL_C35	42a	–	3	–	Negative	Carriage	Strasbourg
SL_C72	42d	–	3	–	Negative	Carriage	Strasbourg
SL_R72	42f	–	*ND*	–	Negative	Deep infection	Rouen
SL_V78	43a	–	3	–	Negative	SSTI	Strasbourg
SL_R41	45b	–	*ND*	–	Negative	SSTI	Rouen
SL_V77	45d	–	7	–	Positive	Catheter related bacteremia	Strasbourg
SL_R29	45d	–	*ND*	–	Positive	Carriage	Rouen
SL_C42	45e	–	7	–	Positive	Carriage	Strasbourg
SL_V31	47a	–	3	–	Negative	Urinary infection	Strasbourg
SL_V10	47c	–	3	–	Negative	Material device infection	Strasbourg
SL_V21	47d	–	3	–	Negative	Catheter related bacteremia	Strasbourg
SL_V48	52a	–	4	–	Negative	Deep infection	Strasbourg
SL_R99	9a	–	*ND*	–	Negative	Carriage	Rouen
SL_T10	9a	–	Singleton	–	Negative	BJI	Rouen
SL_V81	30a	L22STOP	1	–	Negative	SSTI	Strasbourg
SL_V08	40c	L22STOP	1	–	Negative	SSTI	Strasbourg
SL_C60	43c	L22STOP	1	–	Negative	Carriage	Strasbourg
SL_V13	43c	L22STOP	1	–	Negative	BJI on material	Strasbourg
SL_V27	43c	L22STOP	1	–	Negative	BJI on material	Strasbourg
SL_V36	43c	L22STOP	1	–	Negative	BJI	Strasbourg
SL_V66	43c	L22STOP	1	–	Negative	BJI on material	Strasbourg
SL_T13	45f	L22STOP	1	–	Negative	Endocarditis	Rouen
SL_V09	45f	L22STOP	1	–	Negative	SSTI	Strasbourg
SL_V32	45f	L22STOP	1	–	Negative	SSTI	Strasbourg
SL_V64	45f	L22STOP	1	–	Negative	SSTI	Strasbourg
SL_V79	45f	L22STOP	1	–	Negative	Deep infection	Strasbourg
SL_R15	45k	L22STOP	*ND*	–	Negative	Carriage	Rouen
SL_V38	46a	L22STOP	1	–	Negative	BJI on material	Strasbourg
SL_C62	46b	L22STOP	1	–	Negative	Carriage	Strasbourg
SL_V62	46c	L22STOP	1	–	Negative	SSTI	Strasbourg
SL_V33	45a	L22STOP	1	–	Negative	Deep infection	Strasbourg

*The fibrinogen (Fg) binding analysis was performed in two independent experiments measured in at least five wells. Statistical differences compared to *S. aureus* DU5925 (negative control) used Dunnett’s multiple comparisons test (*P* < 0.05). “+++”: *S. lugdunensis* strains that bound significantly to solid-phase Fg (ordinary one-way ANOVA: ^∗∗∗∗^*P* < 0.0001, ^∗∗^*P* < 0.001, and ^∗^*P* < 0.05), “-”: strains with binding inferior to the negative control and “+”: others strains. CC, clonal complex; ND, not determined; SSTI, skin and soft tissue infection; BJI, bone and joint infection.*

Statistical comparisons of the ability of isolates to bind to Fg showed that the group of 19 isolates with the 5′-end deletion of the *fbl* gene was significantly associated with a lower ability to bind to Fg (6.47%) than the group of 36 isolates without the deletion (25.43%) (*P* < 0.001). Moreover, within the group of isolates without the *fbl* deletion, the isolates with ≥43 repeats bound significantly more strongly to Fg (37.64%) than those with <43 repeats (6.23%) (*P* < 0.001). All isolates that significantly adhered to solid-phase Fg were tested positive in the agglutination test. Only three isolates reacted in the agglutination test but did not bind to solid-phase Fg (Table 5). No difference in Fg-binding phenotypes was observed according to the type of clinical specimen.

### *fbl* Gene Expression Analysis

The level of *fbl* gene transcript in mid-exponential phase cells was determined by real-time qRT-PCR for ten isolates selected to represent five Fg-binding-negative and five Fg-binding-positive isolates. Isolates displayed variability in the relative *fbl* expression, ranging from 0.002 to 0.382. However, there was no significant difference (*P* > 0.05) in *fbl* expression level between the two groups (0.015 ± 0.011 for Fg-binding-negative isolates and 0.130 ± 0.172 for Fg-binding-positive isolates).

## Discussion

*Staphylococcus lugdunensis* is a significant human pathogen with distinct clinical and microbiological characteristics compared to other CoNS (Frank et al., 2008; Becker et al., 2014). To better understand the genetic background and population structure of *S. lugdunensis*, several sequence-based methods have been developed, such as MLST, MVLST and more recently TRST (Chassain et al., 2012; Didi et al., 2014; Dahyot et al., 2018). Phylogenetic analysis has shown the clonal population structure and the mutational evolution of this species. Therefore, single *locus* DNA sequencing of the repeated regions of the gene could be a reliable method for the accurate typing of *S. lugdunensis*, as described for *S. aureus* (Shopsin et al., 1999).

The *fbl* R-domain appears to be a reliable marker for typing as it encodes SD-repeat motif. SD repeats have been explored for *S. aureus* genotyping because of their polymorphism in both copy number and sequence (Koreen et al., 2005; Said et al., 2009, 2010). In the present study, we have developed and evaluated a new typing system for *S. lugdunensis* based on the DNA sequencing of the *fbl* R-domain as an alternative technique in clinical and research settings.

Our isolate collection fulfilled van Belkum’s criteria (Van Belkum et al., 2007) for validation and application of typing methods for use in bacterial epidemiology because it included a large “test population” of isolates representative of the genetic diversity of *S. lugdunensis* as well as a set of clinical isolates with well-defined inclusion criteria. Unambiguous *fbl*-types were determined for all the 240 isolates analyzed. The comparison of *fbl*-typing with previously obtained MLST and TRST typing data revealed that *fbl*-typing was highly discriminating compared to MLST, not only in terms of genotypes but also for discriminatory index. In particular, *fbl*-typing showed extensive polymorphism in some clonal groups defined by MLST, like CC1. Moreover, we have shown that there was an excellent correlation between the clustering of isolates by *fbl*-typing and MLST. The success rate of *fbl*-types to predict MLST CCs was 100%, which has allowed us to provide a preliminary guideline for assigning CC from *fbl*-type (Supplementary Table S3). Typing data from future studies could be used to expand our knowledge of *fbl*-MLST mappings, which would be extremely useful in the daily typing of *S. lugdunensis*. Notably, this could be particularly useful to detect putative clonal dissemination of methicillin resistant clones, as described in a tertiary medical center in Taiwan (Cheng et al., 2015).

The fact that *fbl*-typing can group isolates in congruence with MLST indicates that the *fbl locus* is non-recombinogenic in a clonal background. This region is not only variable enough to provide adequate strain discrimination but is also stable enough to group related strains and to be used as a typing tool. Analysis showed that repeat composition and organization, rather than the number of repeats, allowed a correlation between *fbl*-typing and MLST clustering, as described for *spa*-typing of *S. aureus* (Koreen et al., 2004). *fbl*-typing could represent a valuable tool able to simultaneously index genetic variations that accumulate both rapidly (repeat number variations) and slowly (point mutations) by two independent mechanisms. Thus, it could be useful in both long- and short-term epidemiological outbreaks and in population-based studies. Interestingly, *fbl*-typing had the same discriminatory power as TRST, while analyzing a single VNTR. This is probably because *fbl* repeats are more prone to duplication and deletion *via* slipped-strand mispairing as they are smaller (18-bp repeats) than those used for TRST (50 bp on average) (Dahyot et al., 2018).

The major advantage of *fbl*-typing is that a single *locus* allows the retrieval of adequate typing information, relative to MLST and TRST, which require combined allelic information from seven *loci*. The use of a single *locus* marker is less costly, less time-consuming, and less error prone compared to multilocus techniques. Moreover, another advantage of *fbl*-typing is the unambiguity and portability of the sequence data obtained. We have developed an Internet Web site which facilitates information exchange between laboratories and the creation of a large-scale database to evaluate global (such as the study of internationally circulating clones) and local (such as transmission from patient to patient) epidemiology.

However, *fbl*-typing has some limitations. First, from a technical point of view *fbl*-typing may be more difficult than *spa*-typing, even though sequencing technology is improving, due to the large mean size of the amplified region (1180 bp), compared to that of *spa* (556 bp) (Shopsin et al., 1999). Moreover, for some clonal lineages, like CC1 and CC3, we have observed that although they span a wide variety of *fbl*-types, only a few of them seem able to spread efficiently in hospitals. For example, fbl47b was the most predominant *fbl*-type at Strasbourg University Hospital and at Rouen University Hospital, over different study periods. This was also demonstrated in a previous study by alternative typing methods such as TRST (Dahyot et al., 2018). The same phenomenon was described for *S. aureus* with the endemic spread of highly successful *spa* types, resulting in a potential lack of discrimination in local hospital epidemiology (Strommenger et al., 2008). However, the rapid expansion of whole genome sequencing will certainly overcome these limitations. A study has shown that *spa* typing by whole genome sequencing can reliably replace Sanger sequencing (Bartels et al., 2014). Thus, future *fbl*-types of worldwide *S. lugdunensis* isolates will certainly be identified by *in silico* analysis, with the advantage that other genes of interest can easily be analyzed, including genes used for typing like MLST and TRST genes, alternative polymorphic regions, virulence genes and resistance genes. These additional markers could be considered to improve the discriminatory ability of predominant *fbl*-types.

Since Fbl has been shown to be the major Fg-binding protein of *S. lugdunensis* (Marlinghaus et al., 2012), we sought to determine whether the genetic variability of *fbl* observed was related to the ability of the isolates to bind to Fg. Some strains of *S. lugdunensis* could be misidentified as *S. aureus* using agglutination-based kits (Shin et al., 2007). Hence in our study, all isolates were first tested by latex agglutination assay. A positive clumping reaction was observed for 32% of the isolates, which was comparable to that obtained by one study (Szabados et al., 2011), but lower than that reported by others [64.7% (Mateo et al., 2005) and 83.7% (Iorio et al., 2007)]. As previously shown, these differences can be explained by the use of different commercial assays (Personne et al., 1997). Furthermore, even if Fbl was shown as the only Fg-binding surface protein of *S. lugdunensis* (Marlinghaus et al., 2012), other Fg-binding proteins could cross-react in these assays. Indeed, other genes encoding proteins potentially involved in Fg-binding have been described, as a putative Fg/fibronectin binding adhesin which is homologous to FbpA of *S. aureus* (Heilbronner et al., 2011; Szabados et al., 2011; Didi et al., 2014), AtlL (Hussain et al., 2015), and SlsD that has putative structural similarity to the Fg-binding domain of SdrG of *S. epidermidis* (Heilbronner et al., 2011). The ability of 55 isolates to promote adherence to immobilized human Fg showed that binding greatly varied according to the isolates, as described for *S. aureus* (Ythier et al., 2010). These differences in Fg-binding may be explained by several factors.

Our first hypothesis is based on the observation that some isolates revealed a key genetic difference in the 5′-end of the *fbl* gene. A deletion of 4 bp was observed, resulting in a frameshift mutation which potentially radically truncates the Fbl protein from 881 to 21 amino acids. Frameshift mutations tend to occur at repetitive DNA sequences when a misalignment produces either addition or deletion of nucleotides (Lovett, 2004). Here, the deletion occurred in the repeat motif AAAG (Figure 1B). We showed that isolates with this deletion bound to Fg significantly less strongly than the group of isolates without the deletion (*P* < 0.001). To confirm these results, the expression of Fbl at the protein level should be performed by Western-blot analysis from both cellular and supernatant fractions. We predict that for isolates containing a frameshift mutation no Fbl protein will be detected in any cellular fraction. Furthermore, this deletion appeared interestingly to be highly CC-dependent, since it was observed only within CC1 except for one isolate. Most of the isolates belonging to ST6, which is the predicted founder of CC1, presented the deletion. Of note, the deletion was not found for isolates belonging to ST1 and ST7 whereas ST12 and ST15 clones were uniformly associated with the deletion, all four STs evolving from ST6. Therefore, we can assume that this particular event might have arisen in some ST6 strains and has been “transmitted” to ST12 and ST15, constituting a special feature of these STs. This finding has to be confirmed in a larger collection of isolates.

A second explanation could be that the copy number of repeats in the *fbl* R-domain would affect the Fg-binding capacity. This region is thought to span the thick peptidoglycan layer of the cell wall and to allow exposure of the ligand-binding domain at the cell surface by analogy with other Sdr proteins (Hartford et al., 1997). Of note, although major variation exists at the DNA level, the R-domain is mainly constituted of the motif DSDSDA suggesting positive environmental selective pressure and the critical role of serine, aspartate, and alanine in the functionality of the protein. Moreover, many SD repeats are known to be required for functional expression of the ligand-binding domain of ClfA on the cell surface (Hartford et al., 1997). By analogy, it is possible that the length of the repeat domain within *fbl* is critical for protein function. Our findings show that isolates displaying fewer than 43 repeats were significantly weakly adherent to Fg. To confirm this trend and to determine the minimum length required to span the entire cell wall and to display the Fg-binding domain, it would be necessary to construct variants of Fbl with truncated R-domains.

However, some Fg-binding-negative isolates carried more than 43 repeats and did not have the deletion. These results could be explained by the loss or insufficient expression of the *fbl* gene. However, qRT-PCR results were not in favor of this hypothesis. Further analysis should be performed on a larger number of isolates. The last hypothesis is that the production of an extracellular matrix such as a capsule could mask the accessibility of the surface protein (Frank et al., 2008). Risley et al. (2007) described the inhibition of *S. aureus* ClfA-mediated binding to Fg and platelets by capsular polysaccharide expression, which cannot be overcome by a full length repeat region.

The genetic diversity of the *fbl* gene could make it an important target for adaptive evolution through host specialization and other environmental factors. Thus, it could influence the pathogenicity of *S. lugdunensis* by increasing fitness to colonize host surfaces or inducing infective endocarditis. Neither *fbl*-types nor Fg adherence phenotypes predicted clinical contexts. Notably, an infectious endocarditis isolate did not display Fg adhesion while a carrier isolate exhibited very strong adhesion. In *S. aureus*, ClfA was observed to be critical for valve colonization in rats with experimental endocarditis as well as septic arthritis in mice (Josefsson et al., 2001; Que et al., 2005). Conversely, the Fg-binding ability of *S. aureus* strains did not predict the outcome in a rat model of infective endocarditis (Ythier et al., 2010). Authors explained this apparent paradox by a possible differential gene expression *in vivo*, human host factors or adhesin redundancy. In a previous study, it was demonstrated that an *S. lugdunensis* mutant defective in the surface protein Fbl was not significantly less virulent than the wild-type strain in a rat endocarditis model, although a trend toward reduced virulence was observed (Heilbronner et al., 2013). However, only one clinical isolate, that caused a milder course of disease than an *S. aureus* strain, was tested. Therefore, the virulence of other clinical isolates of *S. lugdunensis* and their *fbl* mutants should be compared in animal models of endocarditis but also in murine nasal colonization assays to study the implication of Fbl in adhesion to host surfaces.

## Conclusion

In conclusion, we have demonstrated that the repeat region of *fbl* is polymorphic enough to provide a useful target for the development of a single *locus* genotyping assay. *fbl*-typing is highly discriminating compared to MLST and equivalent to TRST even if only one *locus* is analyzed. Predicting CCs with high accuracy, *fbl*-typing could be a frontline tool for typing *S. lugdunensis* strains in both basic and applied research. The development of a free access Web tool to identify *fbl*-types enables the valuable exchange of information that can be used both in local epidemiology and in international multicenter surveillance of *S. lugdunensis* lineages. Moreover, we have shown that *S. lugdunensis* isolates displayed natural variability in their adherence to immobilized human Fg, which could be partly explained by some genetic variations in the *fbl* gene. It still remains to be determined whether *fbl* polymorphism influences the expression of Fbl protein on the cell surface and has an impact on the colonization of the host tissue and the subsequent development of infection.

## Data Availability

All datasets generated for this study are included in the manuscript and/or the Supplementary Files (http://fbl-typing.univ-rouen.fr/).

## Author Contributions

SD, JL, PF, and MP-C designed the study. XA and GP provided the VISLISI clinical isolates. SD, JL, CD, and FL performed the experiments. SD and JL analyzed the data. SD, JL, and MP-C wrote the manuscript. All authors read and approved the final version of the manuscript.

## Conflict of Interest Statement

The authors declare that the research was conducted in the absence of any commercial or financial relationships that could be construed as a potential conflict of interest.

## Footnotes

1 https://www.ebi.ac.uk/Tools/msa/clustalo/

2 http://fbl-typing.univ-rouen.fr/

3 https://www.graphpad.com/quickcalcs/grubbs2/

## Abbreviations

AW: adjusted Wallace coefficient
CC: clonal complex
CI: confidence interval
CoNS: coagulase-negative *Staphylococcus*
DI: Simpson’s diversity index
Fg: fibrinogen
MLST: multilocus sequence typing
MLVA: multiple-locus VNTR analysis
MVLST: multivirulence-locus sequence typing
PBS: phosphate-buffered saline
ST: sequence type
TRST: tandem repeat sequence typing
TRT: TRST type
VNTR: variable number of tandem repeat
