# Supplementary material for: fbl-Typing of Staphylococcus lugdunensis: A Frontline Tool for Epidemiological Studies, but Not Predictive of Fibrinogen Binding Ability
Source: Front Microbiol. 2019 May 17;10:1109. doi: 10.3389/fmicb.2019.01109 (PMC6533592; doi:10.3389/fmicb.2019.01109)

Supplementary Material

fbl-typing of *Staphylococcus lugdunensis*: a frontline tool for epidemiological studies, but not predictive of fibrinogen binding ability

Sandrine Dahyot*, Jérémie Lebeurre, Floriane Laumay, Xavier Argemi, Charline Dubos, Ludovic Lemée, Gilles Prévost, Patrice François, Martine Pestel-Caron.

*Corresponding author: sandrine.dahyot@chu-rouen.fr

Table S1. Characteristics of the 240 *S. lugdunensis* isolates included in this study.

ND : not determined. [a] N: negative, P: positive

Isolate ID	City	Clinical source	MLST		TRST	*fbl*-type	Premature stop codon in *fbl* gene	Agglutination test [a]
			Sequence type (ST)	Clonal complex (CC)	TRST type (TRT)			
**"TRST panel" (*n* = 128)**								
**SL_V01**	Strasbourg	Deep infection	5	5	10	41a	_	N
**SL_V02**	Strasbourg	Osteoarticular on material	1	1	41	45g	_	P
**SL_V03**	Strasbourg	Skin and soft tissue	3	3	1	47b	_	P
**SL_V04**	Strasbourg	Material device	3	3	1	47b	_	N
**SL_V05**	Strasbourg	Deep infection	1	1	40	45g	_	P
**SL_V06**	Strasbourg	Deep infection	2	2	2	45c	_	N
**SL_V07**	Strasbourg	Deep infection	6	1	8	21a	L22STOP	N
**SL_V08**	Strasbourg	Skin and soft tissue	15	1	47	40c	L22STOP	N
**SL_V09**	Strasbourg	Skin and soft tissue	6	1	8	45f	L22STOP	N
**SL_V10**	Strasbourg	Material device	3	3	1	47c	_	N
**SL_V11**	Strasbourg	Skin and soft tissue	5	5	7	41a	_	N
**SL_V12**	Strasbourg	Skin and soft tissue	5	5	7	41a	_	N
**SL_V13**	Strasbourg	Osteoarticular on material	12	1	3	43c	L22STOP	N
**SL_V14**	Strasbourg	Catheter related bacteremia	24	6	22	41b	_	P
**SL_V15**	Strasbourg	Osteoarticular on material	2	2	16	38a	_	N
**SL_V16**	Strasbourg	Skin and soft tissue	12	1	45	43c	L22STOP	N
**SL_V17**	Strasbourg	Osteoarticular	6	1	21	45f	_	P
**SL_V18**	Strasbourg	Deep infection	24	6	4	41b	_	P
**SL_V19**	Strasbourg	Osteoarticular on material	2	2	2	38b	_	N
**SL_V20**	Strasbourg	Skin and soft tissue	24	6	30	39a	_	P
**SL_V21**	Strasbourg	Catheter related bacteremia	3	3	1	47d	_	N
**SL_V22**	Strasbourg	Endocarditis	24	6	24	42b	_	N
**SL_V23**	Strasbourg	Osteoarticular on material	24	6	26	42c	_	N
**SL_V24**	Strasbourg	Osteoarticular on material	12	1	46	44a	L22STOP	N
**SL_V25**	Strasbourg	Osteoarticular on material	5	5	12	41a	_	N
**SL_V26**	Strasbourg	Skin and soft tissue	3	3	13	47b	_	N
**SL_V27**	Strasbourg	Osteoarticular on material	12	1	33	43c	L22STOP	N
**SL_V28**	Strasbourg	Material device	24	6	5	43b	_	P
**SL_V29**	Strasbourg	Urinary	24	6	5	43b	_	P
**SL_V30**	Strasbourg	Catheter related bacteremia	31	5	25	41a	_	N
**SL_V31**	Strasbourg	Urinary	3	3	1	47a	_	N
**SL_V32**	Strasbourg	Skin and soft tissue	6	1	3	45f	L22STOP	N
**SL_V33**	Strasbourg	Deep infection	6	1	28	45a	L22STOP	N
**SL_V34**	Strasbourg	Skin and soft tissue	4	4	48	52a	_	P
**SL_V35**	Strasbourg	Catheter related bacteremia	1	1	42	45i	_	P
**SL_V36**	Strasbourg	Osteoarticular	12	1	3	43c	L22STOP	N
**SL_V37**	Strasbourg	Endocarditis	1	1	18	45f	_	P
**SL_V38**	Strasbourg	Osteoarticular on material	6	1	20	46a	L22STOP	N
**SL_V39**	Strasbourg	Skin and soft tissue	24	6	5	43b	_	P
**SL_V40**	Strasbourg	Osteoarticular on material	1	1	43	39b	_	P
**SL_V41**	Strasbourg	Skin and soft tissue	3	3	1	47b	_	N
**SL_V42**	Strasbourg	Urinary	24	6	4	40b	_	P
**SL_V43**	Strasbourg	Osteoarticular	24	6	4	40b	_	N
**SL_V44**	Strasbourg	Osteoarticular	6	1	3	39c	_	P
**SL_V45**	Strasbourg	Osteoarticular	24	6	4	41b	_	P
**SL_V46**	Strasbourg	Deep infection	24	6	27	41b	_	N
**SL_V47**	Strasbourg	Osteoarticular	3	3	1	47a	_	N
**SL_V48**	Strasbourg	Deep infection	4	4	49	52a	_	N
**SL_V49**	Strasbourg	Osteoarticular	2	2	2	38a	_	N
**SL_V50**	Strasbourg	Osteoarticular on material	3	3	1	24a	_	N
**SL_V51**	Strasbourg	Osteoarticular	6	1	35	45h	_	P

**SL_V52**	Strasbourg	Osteoarticular on material	1	1	19	35a	_	P
**SL_V53**	Strasbourg	Osteoarticular	2	2	2	45b	_	N
**SL_V54**	Strasbourg	Osteoarticular	5	5	23	41a	_	N
**SL_V55**	Strasbourg	Osteoarticular	31	5	11	41a	_	N
**SL_V56**	Strasbourg	Material device	3	3	17	13a	_	N
**SL_V57**	Strasbourg	Deep infection	1	1	44	21b	_	P
**SL_V58**	Strasbourg	Catheter related bacteremia	4	4	50	49a	_	N
**SL_V59**	Strasbourg	Osteoarticular on material	3	3	1	47b	_	N
**SL_V60**	Strasbourg	Skin and soft tissue	2	2	2	36a	_	N
**SL_V61**	Strasbourg	Osteoarticular on material	3	3	1	47b	_	N
**SL_V62**	Strasbourg	Skin and soft tissue	15	1	36	46c	L22STOP	N
**SL_V63**	Strasbourg	Deep infection	2	2	2	34a	_	N
**SL_V64**	Strasbourg	Skin and soft tissue	6	1	32	45f	L22STOP	N
**SL_V65**	Strasbourg	Osteoarticular	28	Singleton	15	48a	_	P
**SL_V66**	Strasbourg	Osteoarticular on material	12	1	3	43c	L22STOP	N
**SL_V67**	Strasbourg	Skin and soft tissue	3	3	1	45j	_	N
**SL_V68**	Strasbourg	Deep infection	2	2	2	38c	_	N
**SL_V69**	Strasbourg	Urinary	28	Singleton	14	48b	_	P
**SL_V70**	Strasbourg	Skin and soft tissue	27	7	9	41c	_	N
**SL_V71**	Strasbourg	Skin and soft tissue	2	2	2	23a	_	N
**SL_V72**	Strasbourg	Skin and soft tissue	3	3	1	47b	_	N
**SL_V73**	Strasbourg	Urinary	5	5	38	41a	_	P
**SL_V74**	Strasbourg	Bacteremia	2	2	51	45b	_	P
**SL_V75**	Strasbourg	Skin and soft tissue	2	2	2	40a	_	N
**SL_V76**	Strasbourg	Deep infection	3	3	1	47b	_	N
**SL_V77**	Strasbourg	Catheter related bacteremia	26	7	39	45d	_	P
**SL_V78**	Strasbourg	Skin and soft tissue	3	3	1	43a	_	N
**SL_V79**	Strasbourg	Deep infection	6	1	31	45f	L22STOP	N
**SL_V80**	Strasbourg	Osteoarticular on material	6	1	6	45f	_	P
**SL_V81**	Strasbourg	Skin and soft tissue	6	1	6	30a	L22STOP	N
**SL_V82**	Strasbourg	Skin and soft tissue	1	1	34	25a	_	P
**SL_C08**	Strasbourg	Carriage	3	3	1	47b	_	N
**SL_C18**	Strasbourg	Carriage	6	1	29	45f	L22STOP	N
**SL_C21**	Strasbourg	Carriage	3	3	1	47a	_	N
**SL_C22**	Strasbourg	Carriage	3	3	1	47b	_	N
**SL_C27**	Strasbourg	Carriage	3	3	1	47b	L22STOP	N
**SL_C35**	Strasbourg	Carriage	3	3	37	42a	_	N
**SL_C42**	Strasbourg	Carriage	26	7	52	45e	_	P
**SL_C47**	Strasbourg	Carriage	3	3	1	13a	_	N
**SL_C60**	Strasbourg	Carriage	12	1	3	43c	L22STOP	N
**SL_C62**	Strasbourg	Carriage	15	1	6	46b	L22STOP	N
**SL_C68**	Strasbourg	Carriage	2	2	2	38a	_	N
**SL_C71**	Strasbourg	Carriage	27	7	9	41c	_	N
**SL_C72**	Strasbourg	Carriage	3	3	1	42d	_	N
**SL_C75**	Strasbourg	Carriage	3	3	1	47b	_	P
**SL_C77**	Strasbourg	Carriage	3	3	1	47b	_	P
**SL_C85**	Strasbourg	Carriage	3	3	1	13a	_	N
**SL_T02**	Rouen	Endocarditis	8	2	58	45b	_	N
**SL_T03**	Rouen	Bacteremia	9	4	68	23b	_	P
**SL_T09**	Rouen	Bacteremia	5	5	53	42e	_	N
**SL_T10**	Rouen	Osteoarticular	13	Singleton	60	9a	_	N
**SL_T119**	Kronoberg	Carriage	1	1	61	45g	_	P
**SL_T21**	Rouen	Deep infection	10	6	62	42b	_	N
**SL_T27**	Rouen	Osteoarticular on material	4	4	67	49a	_	P
**SL_T37**	Rouen	Catheter related bacteremia	16	3	59	47b	_	N
**SL_T53**	Nantes	Material device	3	3	1	39d	_	N
**SL_T56**	Nantes	Osteoarticular	14	2	2	45b	_	P
**SL_T59**	Nantes	Skin and soft tissue	6	1	64	45f	L22STOP	P
**SL_T62**	Bordeaux	Bacteremia	17	4	66	49a	_	P

**SL_T66**	Bordeaux	Bacteremia	18	5	10	41a	_	N
**SL_T68**	Bordeaux	Bacteremia	7	1	65	44c	_	P
**SL_T73**	Nancy	Bacteremia	19	2	56	42h	_	N
**SL_T74**	Nancy	Skin and soft tissue	12	1	3	43c	L22STOP	P
**SL_T76**	Nancy	Osteoarticular	20	3	57	47b	_	P
**SL_T84**	Montpellier	Deep infection	15	1	69	45f	L22STOP	N
**SL_T85**	Montpellier	Osteoarticular on material	2	2	2	45o	_	N
**SL_AP1**	Rouen	Osteoarticular	3	3	1	47b	_	N
**SL_AP2**	Rouen	Osteoarticular	3	3	1	47b	_	P
**SL_CB1**	Rouen	Bacteremia	15	1	55	46d	L22STOP	N
**SL_CB2**	Rouen	Bacteremia	15	1	55	46d	L22STOP	N
**SL_CJ1**	Rouen	Deep infection	24	6	63	41b	_	P
**SL_CJ2**	Rouen	Bacteremia	24	6	63	41b	_	P
**SL_DJ1**	Rouen	Endocarditis	32	2	2	38a	_	N
**SL_DJ2**	Rouen	Endocarditis	32	2	2	38a	_	N
**SL_LJ1**	Rouen	Deep infection	2	2	2	41d	_	N
**SL_LJ2**	Rouen	Deep infection	2	2	2	41d	_	N
**SL_DSM**		Skin and soft tissue	24	6	54	41b	_	P
			**Other isolates (*n* = 112)**					
**SL_T117**	Kronoberg	Carriage	1	1	*ND*	45f	_	P
**SL_T118**	Kronoberg	Carriage	10	6	*ND*	40b	_	N
**SL_T122**	Tours	Carriage	1	1	*ND*	43g	_	P
**SL_T13**	Rouen	Endocarditis	6	1	*ND*	45f	L22STOP	N
**SL_T29**	Rouen	Material device	1	1	*ND*	43g	_	P
**SL_T55**	Nantes	Skin and soft tissue	10	6	*ND*	41b	_	N
**SL_R01**	Rouen	Carriage	*ND*	*ND*	*ND*	43c	*ND*	N
**SL_R02**	Rouen	Carriage	*ND*	*ND*	*ND*	43c	*ND*	P
**SL_R03**	Rouen	Endocarditis	*ND*	*ND*	*ND*	43c	*ND*	N
**SL_R04**	Rouen	Endocarditis	*ND*	*ND*	*ND*	43c	*ND*	P
**SL_R05**	Rouen	Carriage	*ND*	*ND*	*ND*	45b	*ND*	N
**SL_R06**	Rouen	Carriage	*ND*	*ND*	*ND*	45b	*ND*	P
**SL_R07**	Rouen	Material device	*ND*	*ND*	*ND*	14a	*ND*	N
**SL_R08**	Rouen	Material device	*ND*	*ND*	*ND*	14a	*ND*	N
**SL_R09**	Rouen	Carriage	*ND*	*ND*	*ND*	30b	*ND*	N
**SL_R10**	Rouen	Carriage	*ND*	*ND*	*ND*	30b	*ND*	N
**SL_R11**	Rouen	Bacteremia	*ND*	*ND*	*ND*	47b	_	P
**SL_R12**	Rouen	Carriage	*ND*	*ND*	*ND*	44f	*ND*	P
**SL_R13**	Rouen	Carriage	*ND*	*ND*	*ND*	44d	*ND*	P
**SL_R14**	Rouen	Skin and soft tissue	*ND*	*ND*	*ND*	45f	*ND*	N
**SL_R15**	Rouen	Carriage	*ND*	*ND*	*ND*	45k	L22STOP	N
**SL_R16**	Rouen	Carriage	*ND*	*ND*	*ND*	44e	*ND*	N
**SL_R17**	Rouen	Unknown	*ND*	*ND*	*ND*	41e	*ND*	P
**SL_R18**	Rouen	Carriage	*ND*	*ND*	*ND*	45b	*ND*	N
**SL_R19**	Rouen	Carriage	*ND*	*ND*	*ND*	47b	*ND*	N
**SL_R20**	Rouen	Skin and soft tissue	*ND*	*ND*	*ND*	47b	*ND*	N
**SL_R21**	Rouen	Carriage	*ND*	*ND*	*ND*	45f	*ND*	N
**SL_R22**	Rouen	Carriage	*ND*	*ND*	*ND*	24b	*ND*	N
**SL_R23**	Rouen	Osteoarticular	*ND*	*ND*	*ND*	49a	*ND*	P
**SL_R24**	Rouen	Carriage	*ND*	*ND*	*ND*	43c	*ND*	N
**SL_R25**	Rouen	Osteoarticular	*ND*	*ND*	*ND*	47b	*ND*	N
**SL_R26**	Rouen	Skin and soft tissue	*ND*	*ND*	*ND*	49b	*ND*	P
**SL_R27**	Rouen	Carriage	*ND*	*ND*	*ND*	41a	*ND*	P
**SL_R28**	Rouen	Carriage	*ND*	*ND*	*ND*	42g	*ND*	N
**SL_R29**	Rouen	Carriage	*ND*	*ND*	*ND*	45d	_	P
**SL_R30**	Rouen	Skin and soft tissue	*ND*	*ND*	*ND*	45l	_	P
**SL_R31**	Rouen	Carriage	*ND*	*ND*	*ND*	38a	*ND*	N
**SL_R32**	Rouen	Unknown	*ND*	*ND*	*ND*	41c	*ND*	N
**SL_R33**	Rouen	Carriage	*ND*	*ND*	*ND*	45f	*ND*	P
**SL_R34**	Rouen	Skin and soft tissue	*ND*	*ND*	*ND*	45f	_	P

**SL_R35**	Rouen	Carriage	*ND*	*ND*	*ND*	49c	*ND*	P
**SL_R36**	Rouen	Unknown	*ND*	*ND*	*ND*	43d	*ND*	P
**SL_R37**	Rouen	Carriage	*ND*	*ND*	*ND*	41a	*ND*	N
**SL_R38**	Rouen	Skin and soft tissue	*ND*	*ND*	*ND*	48c	*ND*	N
**SL_R39**	Rouen	Carriage	*ND*	*ND*	*ND*	47b	*ND*	N
**SL_R40**	Rouen	Skin and soft tissue	*ND*	*ND*	*ND*	41b	*ND*	P
**SL_R41**	Rouen	Skin and soft tissue	*ND*	*ND*	*ND*	45b	_	N
**SL_R42**	Rouen	Carriage	*ND*	*ND*	*ND*	47b	*ND*	N
**SL_R43**	Rouen	Unknown	*ND*	*ND*	*ND*	29a	*ND*	N
**SL_R44**	Rouen	Carriage	*ND*	*ND*	*ND*	47b	_	N
**SL_R45**	Rouen	Carriage	*ND*	*ND*	*ND*	47b	*ND*	P
**SL_R46**	Rouen	Carriage	*ND*	*ND*	*ND*	47c	*ND*	N
**SL_R47**	Rouen	Carriage	*ND*	*ND*	*ND*	47b	*ND*	N
**SL_R48**	Rouen	Carriage	*ND*	*ND*	*ND*	46e	*ND*	N
**SL_R49**	Rouen	Carriage	*ND*	*ND*	*ND*	41a	*ND*	N
**SL_R50**	Rouen	Carriage	*ND*	*ND*	*ND*	47b	*ND*	N
**SL_R51**	Rouen	Carriage	*ND*	*ND*	*ND*	45m	*ND*	P
**SL_R52**	Rouen	Carriage	*ND*	*ND*	*ND*	46e	*ND*	N
**SL_R53**	Rouen	Carriage	*ND*	*ND*	*ND*	47b	*ND*	N
**SL_R54**	Rouen	Unknown	*ND*	*ND*	*ND*	45f	*ND*	P
**SL_R55**	Rouen	Unknown	*ND*	*ND*	*ND*	47b	*ND*	N
**SL_R56**	Rouen	Carriage	*ND*	*ND*	*ND*	47b	*ND*	N
**SL_R57**	Rouen	Skin and soft tissue	*ND*	*ND*	*ND*	47b	*ND*	N
**SL_R58**	Rouen	Skin and soft tissue	*ND*	*ND*	*ND*	47b	*ND*	N
**SL_R59**	Rouen	Osteoarticular	*ND*	*ND*	*ND*	13a	_	N
**SL_R60**	Rouen	Skin and soft tissue	*ND*	*ND*	*ND*	45f	*ND*	P
**SL_R61**	Rouen	Skin and soft tissue	*ND*	*ND*	*ND*	46f	*ND*	N
**SL_R62**	Rouen	Skin and soft tissue	*ND*	*ND*	*ND*	42b	*ND*	N
**SL_R63**	Rouen	Skin and soft tissue	*ND*	*ND*	*ND*	43c	*ND*	N
**SL_R64**	Rouen	Skin and soft tissue	*ND*	*ND*	*ND*	41a	*ND*	N
**SL_R65**	Rouen	Skin and soft tissue	*ND*	*ND*	*ND*	36b	*ND*	P
**SL_R66**	Rouen	Deep infection	*ND*	*ND*	*ND*	49a	*ND*	N
**SL_R67**	Rouen	Carriage	*ND*	*ND*	*ND*	49d	*ND*	P
**SL_R68**	Rouen	Carriage	*ND*	*ND*	*ND*	44b	*ND*	P
**SL_R69**	Rouen	Carriage	*ND*	*ND*	*ND*	40b	*ND*	P
**SL_R70**	Rouen	Carriage	*ND*	*ND*	*ND*	45f	*ND*	P
**SL_R71**	Rouen	Carriage	*ND*	*ND*	*ND*	45b	*ND*	N
**SL_R72**	Rouen	Deep infection	*ND*	*ND*	*ND*	42f	_	N
**SL_R73**	Rouen	Carriage	*ND*	*ND*	*ND*	47b	*ND*	N
**SL_R74**	Rouen	Carriage	*ND*	*ND*	*ND*	37a	_	N
**SL_R75**	Rouen	Deep infection	*ND*	*ND*	*ND*	45b	*ND*	P
**SL_R76**	Rouen	Skin and soft tissue	*ND*	*ND*	*ND*	47b	*ND*	N
**SL_R77**	Rouen	Carriage	*ND*	*ND*	*ND*	47b	*ND*	N
**SL_R78**	Rouen	Carriage	*ND*	*ND*	*ND*	46f	*ND*	N
**SL_R79**	Rouen	Carriage	*ND*	*ND*	*ND*	45j	*ND*	N
**SL_R80**	Rouen	Skin and soft tissue	*ND*	*ND*	*ND*	45f	*ND*	N
**SL_R81**	Rouen	Osteoarticular	*ND*	*ND*	*ND*	43e	*ND*	N
**SL_R82**	Rouen	Endocarditis	*ND*	*ND*	*ND*	47b	*ND*	N
**SL_R83**	Rouen	Carriage	*ND*	*ND*	*ND*	41b	*ND*	P
**SL_R84**	Rouen	Carriage	*ND*	*ND*	*ND*	45f	*ND*	N
**SL_R85**	Rouen	Carriage	*ND*	*ND*	*ND*	33a	*ND*	N
**SL_R86**	Rouen	Carriage	*ND*	*ND*	*ND*	45f	*ND*	N
**SL_R87**	Rouen	Carriage	*ND*	*ND*	*ND*	18a	_	N
**SL_R88**	Rouen	Skin and soft tissue	*ND*	*ND*	*ND*	20a	*ND*	N
**SL_R89**	Rouen	Skin and soft tissue	*ND*	*ND*	*ND*	45f	*ND*	P
**SL_R90**	Rouen	Carriage	*ND*	*ND*	*ND*	47b	*ND*	N
**SL_R91**	Rouen	Carriage	*ND*	*ND*	*ND*	47b	*ND*	N
**SL_R92**	Rouen	Deep infection	*ND*	*ND*	*ND*	47b	*ND*	N
**SL_R93**	Rouen	Carriage	*ND*	*ND*	*ND*	52a	*ND*	N

**SL_R94**	Rouen	Carriage	*ND*	*ND*	*ND*	47b	*ND*	N
**SL_R95**	Rouen	Carriage	*ND*	*ND*	*ND*	43f	*ND*	N
**SL_R96**	Rouen	Unknown	*ND*	*ND*	*ND*	47b	*ND*	N
**SL_R97**	Rouen	Carriage	*ND*	*ND*	*ND*	47b	*ND*	N
**SL_R98**	Rouen	Carriage	*ND*	*ND*	*ND*	41b	*ND*	P
**SL_R99**	Rouen	Carriage	*ND*	*ND*	*ND*	9a	_	N
**SL_R100**	Rouen	Carriage	*ND*	*ND*	*ND*	41f	*ND*	N
**SL_R101**	Rouen	Skin and soft tissue	*ND*	*ND*	*ND*	45n	*ND*	N
**SL_R102**	Rouen	Skin and soft tissue	*ND*	*ND*	*ND*	23b	*ND*	N
**SL_R103**	Rouen	Osteoarticular	*ND*	*ND*	*ND*	47b	*ND*	N
**SL_R104**	Rouen	Skin and soft tissue	*ND*	*ND*	*ND*	41g	*ND*	N
**SL_R105**	Rouen	Bacteremia	*ND*	*ND*	*ND*	45b	*ND*	P
**SL_R106**	Rouen	Osteoarticular	*ND*	*ND*	*ND*	45f	*ND*	N

Table S2. DNA and amino acid sequences of the 54 individual *fbl* repeats and their numeric codes identified from the 240 *S. lugdunensis* isolates studied.

Repeat code	DNA sequence	Amino Acid Sequence	Repeat code	DNA sequence	Amino Acid Sequence
0	GATTCCGATAGTGATGCA	DSDSDA	27	GACTCCGACAGTGACGCG	DSDSDA
1	GACTCCGATAGTGATGCA	DSDSDA	28	TATTCCGATAGCGACGCA	YSDSDA
2	GATTCCGACAGTGATGCA	DSDSDA	29	GATTCCGACAGTGACGCA	DSDSDA
3	GATTCCGATAGTGACGCA	DSDSDA	30	GAATCAGATAGCGATGCA	ESDSDA
4	GATTCCGATAGCGATGCA	DSDSDA	31	GACTCCGATAGCGATGCA	DSDSDA
5	GACTCCGATAGTGACGCA	DSDSDA	32	GACTCCGACAGTGATGCA	DSDSDA
6	GATTCCGATAGCGACGCA	DSDSDA	33	GACTTAGATAGTGACGCA	DLDSDA
7	GATTCCGACAGCGATGCA	DSDSDA	34	GATTCCGACAGTGATGCG	DSDSDA
8	GATTCCGACAGCGACGCA	DSDSDA	35	GATTCCGATAGTGATTCA	DSDSDS
9	GACTCCGACAGCGATGCA	DSDSDA	36	GACTCTGACAGTGACGCA	DSDSDA
10	GACTCCGACAGCGACGCA	DSDSDA	37	GACTCCGATAGTAATGCG	DSDSNA
11	GATTCCGATAGTGATGCG	DSDSDA	38	GACTCTGACAGCGATGCG	DSDSDA
12	GACTCCGATAGTGATGCG	DSDSDA	39	GACTCCGATAGTGACGCG	DSDSDA
13	GACTCCGACAGTGATGCG	DSDSDA	40	GATTCAGACAGCGATGCA	DSDSDA
14	GATTCCGACAGCGATGCG	DSDSDA	41	GATTCTGACAGCGACACA	DSDSDT
15	GATTCAGACAGCGATGCG	DSDSDA	42	GACTCCGACAGCGATGTG	DSDSDV
16	GATTCTGACAGTGATGCG	DSDSDA	43	GACTCTGACAGCGACGCA	DSDSDA
17	GACTCTGACAGTGATGCG	DSDSDA	44	GACTCAGATAGTGATTCA	DSDSDS
18	GACTCAGATAGTGACGCA	DSDSDA	45	GATTCTGACAGCAATTCC	DSDSNS
19	GACTCAGATAGCGATGCA	DSDSDA	46	GGTTCCGACAGCAATGTG	GSDSNV
20	GACTCAGATAGCGACGCA	DSDSDA	47	GACTCAGACAGCGACGCA	DSDSDA
21	GATTCTGACAGCGACGCA	DSDSDA	48	GACTCAGATAGCGATGCG	DSDSDA
22	GATTCAGACAGCGACGCA	DSDSDA	49	GACACAGATAGTGACGCA	DTDSDA
23	GACTCCGACAGCGATGCG	DSDSDA	50	AATTCCGATAGTGATGCA	NSDSDA
24	GACTCCGACAGTGACGCA	DSDSDA	51	GAGTCAGATAGCGATGCA	ESDSDA
25	GACTCAGATAGTGATGCA	DSDSDA	52	GATTCCGACAGTGACGCG	DSDSDA
26	GACTCCGATAGCGACGCA	DSDSDA	53	GCATGGGATAGTGATGCA	AWDSDA

Table S3. List of the 92 *fbl-* types identified from the 240 *S. lugdunensis* isolates studied, and preliminary guideline for the assignment of MLST clonal complexes (CCs) using *fbl-* typing.

CC*: CC predicted from the repeat alignment of the *fbl-* types.

ST: sequence type

fbl- type	*fbl* cluster	Repeat succession	CC	ST
**fbl21a**	**_**	0-6-13-1-7-18-2-11-17-12-13-5-14-10-0-30-6-6-45-46-44	CC1	ST6
**fbl21b**	**_**	0-6-13-1-7-18-2-11-17-12-13-5-4-18-16-20-9-6-45-46-44	CC1	ST1
**fbl25a**	**_**	0-6-13-1-7-18-2-11-17-12-13-5-14-10-0-19-6-11-8-0-9-6-45-46-44	CC1	ST1
**fbl29a**	**_**	0-6-13-1-7-18-2-11-17-12-13-5-14-10-0-19-6-11-8-0-4-18-16-20-9-6-45-46-44	CC1*	_
**fbl30a**	**_**	0-6-13-1-7-18-2-11-38-10-0-19-6-11-8-0-9-6-21-0-10-19-18-16-20-9-6-45-46-44	CC1*	_
**fbl35a**	**_**	0-6-13-1-7-18-2-11-17-12-13-5-14-10-0-19-6-21-1-10-19-18-16-20-16-19-15-19-22-2-3-4-18-16-44	CC1	ST1
**fbl39b**	**_**	0-6-13-1-7-1-7-18-14-10-0-19-6-11-8-0-9-6-21-0-10-19-18-16-20-16-19-15-19-8-2-18-16-20-9-6-45-46-44	CC1	ST1
**fbl39c**	**_**	0-7-18-2-11-17-12-13-5-14-10-19-18-16-20-16-20-0-9-19-18-16-20-16-19-15-19-22-2-3-4-18-16-20-9-6-45-46-44	CC1	ST6
**fbl40c**	**_**	0-11-17-12-13-5-14-10-0-19-6-11-8-0-9-6-21-0-10-19-18-16-20-16-19-15-19-22-2-3-4-18-16-20-16-19-6-45-46-44	CC1	ST15
**fbl41e**	**1**	0-6-13-1-7-18-34-12-13-5-14-10-0-19-6-11-10-0-9-6-21-0-10-19-18-16-19-15-19-22-2-3-4-18-16-20-9-6-45-46-44	CC1*	_
**fbl41g**	**1**	0-6-13-1-7-18-2-11-17-12-13-5-14-10-0-19-6-11-8-0-9-6-21-0-10-19-18-16-20-16-19-15-19-22-34-20-9-6-45-46-44	CC1*	_
**fbl43c**	**1**	0-6-13-1-7-18-2-11-17-12-13-5-14-10-0-19-6-11-8-0-9-6-21-0-10-19-18-16-19-15-19-22-2-3-4-18-16-20-9-6-45-46-44	CC1	ST12
**fbl43e**	**1**	0-6-13-1-7-18-2-11-17-12-13-5-14-10-0-19-6-11-8-0-9-6-21-0-10-19-18-16-19-15-19-22-2-3-10-18-16-20-9-6-45-46-44	CC1*	_
**fbl43g**	**_**	0-6-13-5-7-18-2-11-17-12-13-5-14-10-0-19-6-11-8-0-9-6-21-1-10-19-18-16-20-16-19-15-19-22-2-3-4-18-16-20-45-46-44	CC1	ST1
**fbl44a**	**1**	0-6-13-1-7-18-2-11-17-12-13-5-14-10-0-19-6-6-11-8-0-9-6-21-0-10-19-18-16-19-15-19-22-2-3-4-18-16-20-9-6-45-46-44	CC1	ST12
**fbl44b**	**1**	0-6-13-1-7-18-2-11-17-13-5-14-10-0-19-6-11-10-0-9-6-21-0-10-19-18-16-20-16-19-15-19-22-2-3-4-18-16-20-9-6-45-46-44	CC1*	_
**fbl44c**	**1**	0-6-13-1-7-18-2-0-11-13-5-14-10-0-19-6-11-8-0-9-6-21-0-10-19-18-16-20-16-19-15-19-22-2-3-4-18-16-20-9-6-45-46-44	CC1	ST7
**fbl44d**	**1**	0-6-13-1-7-18-2-11-12-13-5-14-10-0-19-6-11-8-0-9-6-21-0-10-19-18-16-20-16-19-15-19-22-2-3-4-18-16-20-9-6-45-46-44	CC1*	_
**fbl44e**	**1**	0-6-13-1-7-18-2-11-17-12-13-5-14-10-0-6-11-8-0-9-6-21-0-10-19-18-16-20-16-19-15-19-22-2-3-4-18-16-20-9-6-45-46-44	CC1*	_
**fbl44f**	**1**	6-13-1-7-18-2-11-17-12-13-5-14-10-0-19-6-11-8-0-9-6-21-0-10-19-18-16-20-16-19-15-19-22-2-3-4-18-16-20-9-6-45-46-44	CC1*	_
**fbl45a**	**1**	0-6-13-1-7-18-2-11-17-13-13-5-14-10-0-19-6-11-8-0-9-6-21-0-10-19-18-16-20-16-19-15-19-22-2-3-4-18-16-20-9-6-45-46-44	CC1	ST6
**fbl45f**	**1**	0-6-13-1-7-18-2-11-17-12-13-5-14-10-0-19-6-11-8-0-9-6-21-0-10-19-18-16-20-16-19-15-19-22-2-3-4-18-16-20-9-6-45-46-44	CC1	ST1, ST6, ST15
**fbl45g**	**1**	0-6-13-1-7-18-2-11-17-12-13-5-14-10-0-19-6-11-8-0-9-6-21-1-10-19-18-16-20-16-19-15-19-22-2-3-4-18-16-20-9-6-45-46-44	CC1	ST1
**fbl45h**	**1**	0-6-13-1-7-18-2-11-17-12-13-5-14-43-0-19-6-11-8-0-9-6-21-0-10-19-18-16-20-16-19-15-19-22-2-3-4-18-16-20-9-6-45-46-44	CC1	ST6
**fbl45i**	**1**	0-6-13-1-7-18-2-11-17-12-13-5-14-10-0-19-6-11-8-0-19-6-21-0-10-19-18-16-20-16-19-15-19-22-2-3-4-18-16-20-9-6-45-46-44	CC1	ST1
**fbl45k**	**1**	0-6-13-1-7-49-2-11-17-12-13-5-14-10-0-19-6-11-8-0-9-6-21-0-10-19-18-16-20-16-19-15-19-22-2-3-4-18-16-20-9-6-45-46-44	CC1*	_
**fbl45l**	**1**	0-6-13-1-7-18-2-11-17-12-13-5-14-10-0-19-6-11-8-0-9-6-21-0-10-19-18-16-20-16-19-15-19-22-2-5-4-18-16-20-9-6-45-46-44	CC1*	_
**fbl45m**	**1**	0-6-13-1-7-18-2-11-17-12-13-5-14-10-0-19-6-11-8-0-9-6-21-0-10-19-18-16-20-16-19-15-19-8-2-3-4-18-16-20-9-6-45-46-44	CC1*	_
**fbl45n**	**_**	0-6-13-1-7-18-2-11-17-12-13-5-14-10-0-31-6-11-8-0-9-6-21-0-10-19-18-16-20-16-19-15-19-22-2-3-4-18-16-20-9-6-45-46-44	CC1*	_
**fbl46a**	**1**	0-6-13-1-7-18-2-11-17-12-13-5-14-10-0-19-6-11-8-0-9-6-21-0-10-19-18-16-20-16-19-15-19-22-2-3-3-4-18-16-20-9-6-45-46-44	CC1	ST6
**fbl46b**	**1**	0-6-13-1-7-18-2-2-11-17-12-13-5-14-10-0-19-6-11-8-0-9-6-21-0-10-19-18-16-20-16-19-15-19-22-2-3-4-18-16-20-9-6-45-46-44	CC1	ST15
**fbl46c**	**8**	0-6-13-1-7-18-2-11-17-12-13-5-14-10-0-19-6-13-8-0-9-6-21-0-10-19-18-16-20-16-19-15-19-22-2-3-4-18-16-20-16-19-6-45-46-44	CC1	ST15
**fbl46d**	**8**	0-6-13-1-7-18-2-11-17-12-13-5-14-10-0-19-6-11-8-0-9-6-21-0-10-19-18-16-20-16-19-15-19-8-2-3-4-18-16-20-16-19-6-45-46-44	CC1	ST15
**fbl46e**	**8**	0-6-13-1-7-18-2-11-17-12-13-5-14-10-0-19-6-11-8-0-9-6-21-0-10-19-18-16-20-16-19-15-19-22-2-3-4-18-16-20-16-19-6-45-46-44	CC1*	_
**fbl46f**	**8**	0-6-13-1-7-18-2-11-17-12-13-5-14-10-0-19-6-11-10-0-9-6-21-0-10-19-18-16-20-16-19-15-19-22-2-3-4-18-16-20-16-19-6-45-46-44	CC1*	_
**fbl23a**	**_**	0-28-20-23-1-7-5-7-11-26-12-0-6-20-23-9-11-13-13-26-45-46-44	CC2	ST2
**fbl30b**	**_**	0-28-20-23-1-7-26-23-12-1-7-18-7-11-13-18-23-10-0-19-8-19-9-11-13-13-26-45-46-44	CC2*	_
**fbl33a**	**6**	0-28-20-23-12-1-7-18-7-11-13-18-23-10-0-19-3-0-27-29-0-18-27-7-8-19-9-11-13-26-45-46-44	CC2*	_
**fbl34a**	**6**	0-28-20-23-12-1-7-18-7-11-13-18-23-10-0-19-3-0-27-29-0-18-7-7-8-19-9-11-13-13-26-45-46-44	CC2	ST2
**fbl36a**	**6**	0-28-20-23-1-7-26-23-12-1-7-18-7-11-13-18-23-10-0-19-3-0-27-29-0-18-7-8-19-9-11-13-26-45-46-44	CC2	ST2
**fbl38a**	**6**	0-28-20-23-1-7-26-23-12-1-7-18-7-11-13-18-23-10-0-19-3-0-27-29-0-18-7-7-8-19-9-11-13-13-26-45-46-44	CC2	ST2, ST32
**fbl38b**	**6**	0-28-20-23-1-7-26-23-12-1-7-18-7-11-13-18-23-10-0-19-3-0-27-29-0-33-7-7-8-19-9-11-13-13-26-45-46-44	CC2	ST2
**fbl38c**	**6**	0-7-26-23-1-7-26-23-12-1-7-18-7-11-13-18-23-10-0-19-3-0-27-29-0-18-7-7-8-19-9-11-13-13-26-45-46-44	CC2	ST2
**fbl40a**	**5**	0-28-20-23-26-12-0-6-20-23-12-1-7-18-7-11-13-18-23-10-0-19-3-0-27-29-0-18-7-7-8-19-9-11-13-13-26-45-46-44	CC2	ST2
**fbl41d**	**_**	0-28-20-23-1-7-5-7-11-26-12-0-6-20-23-12-1-7-18-23-10-0-19-3-0-27-29-0-18-7-7-8-19-9-11-13-13-26-45-46-44	CC2	ST2
**fbl42h**	**_**	0-28-20-23-1-7-5-7-11-26-12-0-6-20-23-12-1-7-18-7-11-13-18-23-10-0-19-3-0-27-29-7-7-8-19-9-11-13-26-45-46-44	CC2	ST19
**fbl45b**	**5**	0-28-20-23-1-7-5-7-11-26-12-0-6-20-23-12-1-7-18-7-11-13-18-23-10-0-19-3-0-27-29-0-18-7-7-8-19-9-11-13-13-26-45-46-44	CC2	ST2, ST8, ST14
**fbl45c**	**5**	0-28-20-23-1-7-5-7-11-26-12-0-6-20-9-12-1-7-18-7-11-13-18-23-10-0-19-3-0-27-29-0-18-7-7-8-19-9-11-13-13-26-45-46-44	CC2	ST2
**fbl45o**	**_**	0-28-20-23-1-7-18-7-11-26-12-0-6-20-23-12-1-7-18-7-11-13-18-23-10-0-19-3-0-27-29-0-18-7-7-8-19-9-11-13-13-26-45-46-44	CC2	ST2
**fbl13a**	**_**	0-6-20-23-12-1-7-20-7-18-14-26-44	CC3	ST3
**fbl18a**	**_**	0-6-20-23-12-1-7-20-7-11-13-18-14-10-0-11-26-44	CC3*	_
**fbl24a**	**_**	0-6-18-7-8-20-23-25-7-8-20-23-25-7-8-19-23-25-7-7-18-14-26-44	CC3	ST3
**fbl24b**	**_**	50-6-20-23-12-1-7-20-7-8-20-23-25-7-8-19-23-25-7-7-18-14-26-44	CC3*	_
**fbl37a**	**_**	0-6-20-23-12-1-7-20-7-11-13-18-14-10-0-11-9-11-8-0-23-1-27-21-1-18-7-8-20-23-25-7-7-18-14-26-44	CC3*	_
**fbl39d**	**_**	0-6-20-23-12-1-7-20-7-11-13-18-14-10-52-21-1-18-7-8-20-23-25-7-8-20-23-25-7-8-19-23-25-7-7-18-14-26-44	CC3	ST3
**fbl42a**	**_**	0-6-20-23-12-1-7-20-7-11-13-18-14-10-0-11-9-0-27-21-1-18-7-8-20-23-25-8-20-23-25-7-8-19-23-25-7-7-18-14-26-44	CC3	ST3
**fbl42d**	**_**	0-6-20-23-12-1-7-20-7-11-13-18-14-10-0-11-9-11-8-0-23-1-27-21-1-18-7-8-20-23-25-7-8-20-23-25-7-8-19-23-26-44	CC3	ST3
**fbl42f**	**_**	0-6-20-23-12-1-7-20-7-11-13-18-14-10-0-11-9-11-8-0-23-1-27-21-1-25-7-8-20-23-25-7-8-19-23-25-7-7-18-14-26-44	CC3*	_
**fbl42g**	**_**	0-6-20-7-11-13-18-14-10-0-11-9-11-8-0-23-1-27-21-1-18-7-8-20-23-25-7-8-20-23-25-7-8-19-23-25-7-7-18-14-26-44	CC3*	_
**fbl43a**	**_**	0-6-20-23-12-1-7-20-7-11-13-18-14-10-0-11-9-11-8-0-23-1-27-21-1-18-7-8-47-20-23-25-7-8-19-23-25-7-7-18-14-26-44	CC3	ST3
**fbl45j**	**2**	0-6-20-23-12-1-7-20-7-11-13-18-14-10-0-11-9-11-8-0-23-1-27-21-1-18-7-8-20-23-25-7-8-20-23-25-7-8-19-23-25-7-7-26-44	CC3	ST3
**fbl47a**	**2**	0-6-20-23-12-1-7-20-7-11-13-18-14-10-0-11-9-11-8-0-23-1-27-21-1-18-7-8-20-14-25-7-8-20-23-25-7-8-19-23-25-7-7-18-14-26-44	CC3	ST3
**fbl47b**	**2**	0-6-20-23-12-1-7-20-7-11-13-18-14-10-0-11-9-11-8-0-23-1-27-21-1-18-7-8-20-23-25-7-8-20-23-25-7-8-19-23-25-7-7-18-14-26-44	CC3	ST3, ST16, ST20
**fbl47c**	**2**	0-6-20-23-12-1-7-20-7-11-13-18-14-10-0-11-9-11-8-0-23-1-27-21-1-18-7-8-20-23-25-7-8-20-23-1-7-8-19-23-25-7-7-18-14-26-44	CC3	ST3
**fbl47d**	**2**	0-6-20-10-11-1-7-20-7-11-13-18-14-10-0-11-9-11-8-0-23-1-27-21-1-18-7-8-20-23-25-7-8-20-23-25-7-8-19-23-25-7-7-18-14-26-44	CC3	ST3
**fbl48c**	**2**	0-6-20-20-23-12-1-7-20-7-11-13-18-14-10-0-11-9-11-8-0-23-1-27-21-1-18-7-8-20-23-25-7-8-20-23-25-7-8-19-23-25-7-7-18-14-26-44	CC3*	_
**fbl23b**	**_**	53-23-1-9-11-13-12-13-5-14-10-0-31-6-14-1-27-2-11-20-45-46-44	CC4	ST9
**fbl49a**	**7**	0-6-23-1-7-18-7-11-13-13-12-5-14-10-0-19-6-11-8-0-23-1-27-2-11-20-21-1-8-19-34-19-7-21-11-19-9-21-2-3-4-18-16-20-9-6-45-46-44	CC4	ST4 , ST17
**fbl49b**	**7**	0-6-23-1-7-18-7-11-13-13-12-5-14-10-0-19-6-11-8-0-23-1-27-2-0-20-21-1-8-19-34-19-7-21-11-19-9-21-2-3-4-18-16-20-9-6-45-46-44	CC4*	_
**fbl49c**	**7**	0-6-23-1-7-18-7-11-13-13-12-5-14-10-0-51-6-11-8-0-23-1-27-2-11-20-21-1-8-19-34-19-7-21-11-19-9-21-2-3-4-18-16-20-9-6-45-46-44	CC4*	_
**fbl49d**	**7**	0-6-23-1-7-18-7-11-13-13-13-5-14-10-0-19-6-11-8-0-23-1-27-2-11-20-21-1-8-19-34-19-7-21-11-19-9-21-2-3-4-18-16-20-9-6-45-46-44	CC4*	_
**fbl52a**	**7**	0-6-23-1-6-23-1-7-18-7-11-13-13-12-5-14-10-0-19-6-11-8-0-23-1-27-2-11-20-21-1-8-19-34-19-7-21-11-19-9-21-2-3-4-18-16-20-9-6-45-46-44	CC4	ST4
**fbl14a**	**_**	0-6-20-23-1-14-20-9-11-13-26-45-46-44	CC5*	_
**fbl20a**	**_**	0-11-20-24-0-18-8-20-23-25-7-8-19-9-11-13-26-45-46-44	CC5*	_
**fbl41a**	**4**	0-6-20-23-1-14-20-9-11-13-5-14-10-0-19-6-7-8-14-13-5-7-11-20-24-0-18-8-20-23-25-7-8-19-9-11-13-26-45-46-44	CC5	ST5, ST18, ST31
**fbl41f**	**_**	0-6-20-23-1-14-20-9-11-13-5-14-9-0-25-6-4-7-4-13-5-7-11-20-24-0-18-8-20-23-25-7-8-19-9-11-13-26-45-46-44	CC5*	_
**fbl42e**	**4**	0-6-20-23-1-14-20-9-11-13-5-14-10-0-19-6-7-8-14-13-5-7-11-20-24-0-18-8-20-23-25-7-8-19-9-9-11-13-26-45-46-44	CC5	ST5
**fbl36b**	**_**	0-6-23-31-6-11-8-0-23-1-9-11-13-12-13-5-14-10-0-31-6-14-32-27-2-11-20-21-1-8-20-31-6-45-46-44	CC6*	_
**fbl39a**	**_**	0-6-23-31-6-11-8-0-42-1-9-11-13-13-5-14-10-0-31-6-2-27-2-11-20-21-1-8-20-31-4-18-16-20-9-6-45-46-44	CC6	ST24
**fbl40b**	**3**	0-6-23-31-6-11-8-0-23-1-9-11-13-12-13-5-14-10-0-31-6-14-32-27-2-11-20-21-1-8-20-31-18-16-20-9-6-45-46-44	CC6	ST10, ST24
**fbl41b**	**3**	0-6-23-31-6-11-8-0-23-1-9-11-13-12-13-5-14-10-0-31-6-14-32-27-2-11-20-21-1-8-20-31-4-18-16-20-9-6-45-46-44	CC6	ST10, ST24
**fbl42b**	**3**	0-6-23-31-6-11-8-0-23-1-9-11-13-12-13-5-14-10-0-31-4-6-14-32-27-2-11-20-21-1-8-20-31-4-18-16-20-9-6-45-46-44	CC6	ST10, ST24
**fbl42c**	**3**	0-6-23-31-6-11-8-0-23-1-9-11-32-12-13-5-14-10-0-31-4-6-14-32-27-2-11-20-21-1-8-20-31-4-18-16-20-9-6-45-46-44	CC6	ST24
**fbl43b**	**3**	0-6-23-31-6-11-8-6-11-8-0-23-1-9-11-13-13-5-14-10-0-31-6-14-32-27-2-11-20-21-1-8-20-31-4-18-16-20-9-6-45-46-44	CC6	ST24
**fbl43d**	**10**	0-6-23-31-6-11-8-6-11-8-0-23-1-23-8-0-23-1-9-11-13-13-5-14-32-27-2-11-20-21-1-8-20-31-4-18-16-20-9-6-45-46-44	CC6*	_
**fbl43f**	**10**	0-6-23-31-6-11-8-6-11-8-0-23-1-23-7-0-23-1-9-11-13-13-5-14-32-27-2-11-20-8-1-8-20-31-4-18-16-20-9-6-45-46-44	CC6*	_
**fbl41c**	**_**	0-6-13-37-5-14-10-0-19-6-18-7-0-8-19-34-19-7-22-11-18-4-18-16-20-16-19-15-19-22-2-3-4-18-16-20-9-6-45-46-44	CC7	ST27
**fbl45d**	**9**	0-6-23-39-0-23-1-27-2-11-26-6-23-12-13-5-14-10-0-31-6-0-10-5-14-10-1-8-20-31-11-19-40-41-2-3-4-18-16-19-9-6-45-46-44	CC7	ST26
**fbl45e**	**9**	0-6-23-39-0-23-1-27-2-11-26-6-23-12-13-5-7-10-0-31-6-0-10-5-14-10-1-8-20-31-11-19-40-41-2-3-4-18-16-19-9-6-45-46-44	CC7	ST26
**fbl48a**	**_**	0-6-20-23-1-14-20-9-11-5-14-10-0-20-6-0-7-14-13-1-24-0-18-24-0-18-24-35-18-7-7-18-36-7-10-20-23-1-7-8-19-9-11-13-6-45-46-44	singleton	ST28
**fbl48b**	**_**	0-6-20-23-1-14-20-9-11-5-14-10-0-19-6-0-7-14-13-1-24-0-18-24-0-5-24-35-18-7-7-18-36-7-10-20-23-25-7-8-19-9-11-13-6-45-46-44	singleton	ST28
**fbl9a**	**_**	0-6-48-23-1-7-18-4-44	singleton	ST13

Figure S1. Adherence of 55 *S. lugdunensis* isolates to immobilized fibrinogen. *S. aureus* 8325-was used as positive control (adherent); *S. aureus* DU5925 was used as a negative one (non-adherent). Adherence of isolates was expressed relative to that of 8325-4 (100%). Results were expressed as mean percentages +/- standard error of mean. Statistical differences compared to DU5925 used Dunnett's multiple comparisons test ($P < 0.05$). Ordinary one-way ANOVA: $P < 0.0001$ ****.

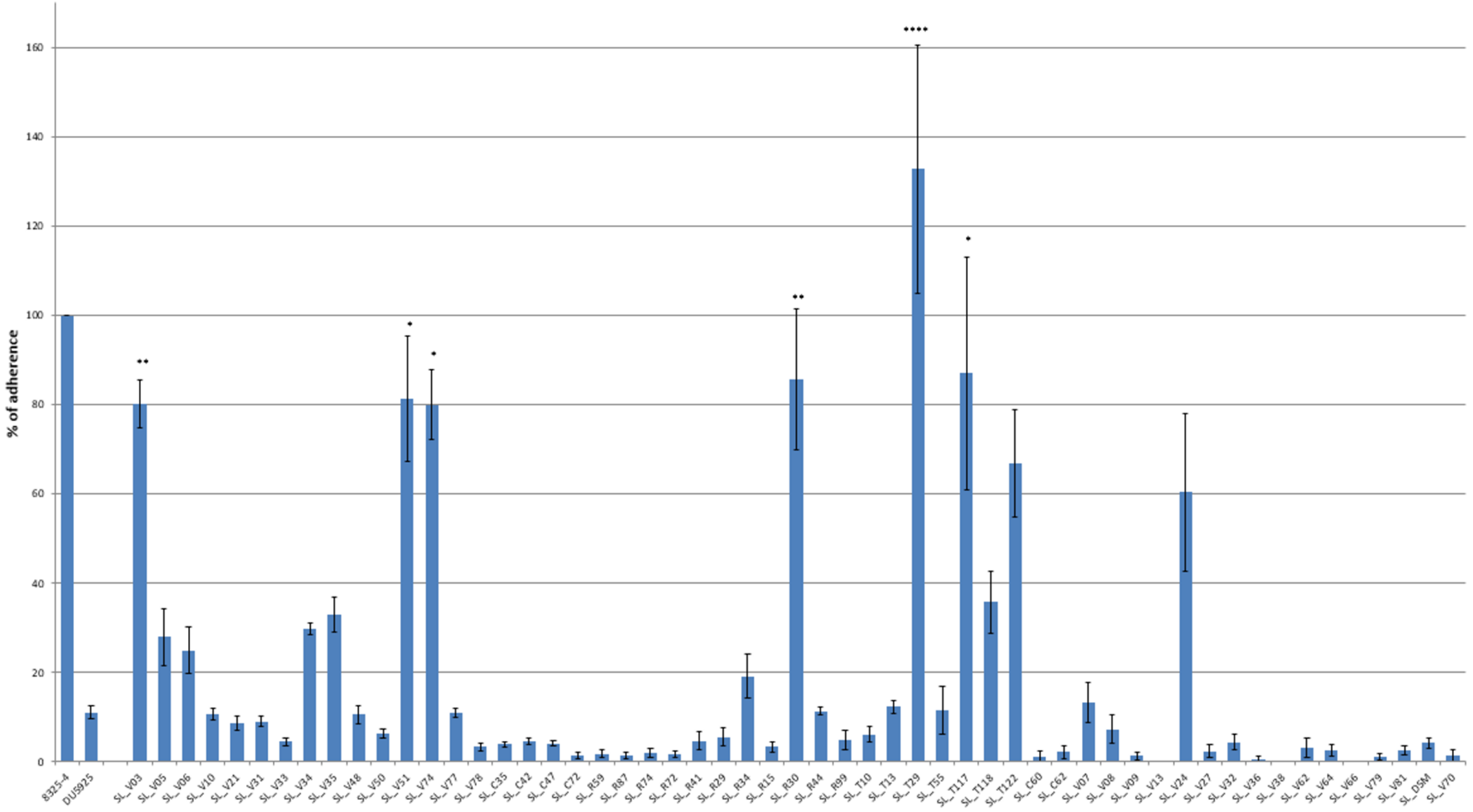

Supplement: Supplementary file 1 [file Data_Sheet_1.PDF]
